# Ultrasound surveillance for deep venous thrombosis and subsequent venous thromboembolism in adults with trauma: A systematic review and meta-analysis

**DOI:** 10.1097/MD.0000000000035625

**Published:** 2023-10-27

**Authors:** Abdulaziz M. Al-Sharydah, Mohammed S. Alshahrani, Khalid Maghrabi, Wail Tashkandi, Marwa Amer

**Affiliations:** a Diagnostic and Interventional Radiology Department, King Fahd Hospital of the University, Imam Abdulrahman Bin Faisal University, Dammam, Saudi Arabia; b Department of Emergency and Critical Care, King Fahd Hospital of the University, Imam Abdulrahman Bin Faisal University, Dammam, Saudi Arabia; c Department of Critical Care Medicine, King Faisal Specialist Hospital and Research Center, Riyadh, Saudi Arabia; d Department of Surgery, King Abdulaziz University, Jeddah, Saudi Arabia; e Department of Critical Care, Fakeeh Care Group, Jeddah, Saudi Arabia; f Medical/Critical Pharmacy Division, King Faisal Specialist Hospital and Research Center, Riyadh, Saudi Arabia; g College of Medicine and Pharmacy, Alfaisal University, Riyadh, Saudi Arabia.

**Keywords:** proximal deep vein thrombosis, pulmonary embolism, trauma, ultrasound surveillance, venous thromboembolism

## Abstract

**Background:**

Studies have shown routine ultrasound surveillance (RUSS) will facilitate deep vein thrombosis (DVT) detection in patients with trauma and reduce the subsequent incidence of pulmonary embolism (PE); however, the findings were inconsistent. In adults with trauma at a high risk of venous thromboembolism, this systematic review and meta-analysis compared RUSS outcomes with those of “no RUSS.”

**Methods:**

Three databases were screened for relevant articles from inception to October 18, 2021. Randomized controlled trials (RCTs) and observational studies comparing RUSS with no RUSS were included. We used relative risks (RRs), odds ratios (ORs), and mean differences to pool effect estimates for dichotomous and continuous outcomes. The cochrane risk of bias or the risk of bias in non-randomized studies of interventions were used to assess bias risk. The grading of recommendations, assessment, development, and evaluation framework assessed the certainty of the evidence.

**Findings:**

Out of 1685 articles, 5 met the inclusion criteria (RCT: 1; observational studies: 4). Observational studies suggested RUSS is associated with higher odds of DVT detection (OR, 4.87; 95% confidence interval [CI], 3.13–7.57; very low certainty). Whereas higher risks of DVT were associated with RUSS in the RCT (distal DVT: RR, 15.48; 95% CI, 7.62–31.48; low certainty, and proximal DVT: RR, 2.37; 95% CI, 1.04–5.39; very low certainty). Reduced odds of PE risk were observed with the RUSS (OR, 0.47; 95% CI, 0.24–0.91; very low certainty). Observational studies indicated that RUSS had an uncertain effect on mortality (OR, 0.46; 95% CI, 0.06–3.49). In the RCT, times to proximal and distal DVT diagnoses were shorter with RUSS (proximal DVT, mean difference 2.25 days shorter [95% CI, 5.74–1.24]; distal DVT, mean differences 1.56 days shorter [95% CI, 4.22–1.12]; very low certainty for both). Increasing bleeding risk was not linked to the RUSS group (RR, 1.24; 95% CI, 0.31–4.92).

**Interpretation:**

The RUSS efficacy in adults with trauma at high risk for venous thromboembolism showed that it increases DVT detection, decreases PE incidence, and shortens the time to DVT diagnosis, with an uncertain impact on mortality. The evidence is low or very low in certainty because of bias, inconsistency, imprecision, and indirectness.

## 1. Introduction

Major trauma is a significant risk factor for deep vein thrombosis (DVT) and pulmonary embolism (PE). The incidence of venous thromboembolism (VTE) varies among patients with trauma. The estimated incidence of distal DVT is 58%, while the estimated incidence of proximal DVT ranges from 14.7% to 27.3% (depending on the test used for diagnosing lower extremity DVT [LE DVT] as well as the mechanism of injury).^[1,2]^ The mortality rates among patients with DVT are 3% to 9%.^[3–6]^

There is no consensus regarding the concept and practice of ultrasound (US) examination for LE DVT.^[2,7–11]^ US is a noninvasive modality, and routine ultrasound surveillance (RUSS) can aid in the detection of asymptomatic or silent DVT. This in turn can enable the initiation of optimal anticoagulant therapies to preclude the progression of DVT to PE, thereby preventing the occurrence of major VTE sequelae (such as recurrent VTE and post-thrombotic syndrome).^[2,3,12,13]^

However, there are inconsistencies in the reported effects of RUSS on the mortality and PE detection rates in patients with DVT. Some observational studies have reported a reduction in PE detection rates, while others have revealed no changes or even an increase in PE detection and mortality rates.

In terms of clinical efficiency, some studies have shown the clinical value and necessity of RUSS for patients with trauma who are at a high risk of VTE development, especially those with active bleeding (which prevents early initiation of pharmacological VTE prophylaxis or leads to its frequent interruption) and those with orthopedic injuries and fixation devices (which prevent initiation of mechanical prophylaxis).^[2,9,10,14–16]^ The utility and cost-effectiveness of LE US-based screening protocols for patients with trauma differ among trauma centers and remain controversial, particularly in terms of the eligible patients, screening frequency, and whether RUSS influences clinically important VTE outcomes.^[3,17–19]^ Thus, this systematic review and meta-analysis (SRMA) was conducted as part of a larger clinical practice guideline evaluating VTE prophylaxis in trauma patients that was issued recently by the Saudi Critical Care Society^[20]^ and endorsed by the Scandinavian Society of Anesthesiology and Intensive Care Medicine.^[21]^ The essential notion behind conducting this SRMA was to provide the best available evidence tackling if we should recommend versus not to recommend a RUSS in at-risk trauma patients who are not candidates for pharmacological VTE prophylaxis. Despite the available intuitive evidence, the impact of RUSS on preventing the propagation of DVT into PE or fatal PE appears to be inconsistent, and the frequency of recommended RUSS is contradictory. These intuitions neither applied to ambulating or low-risk trauma patients nor patients with signs or symptoms suggestive of DVT in whom RUSS is indicated.

## 2. Methods

We developed a predefined protocol for the present SRMA on October 27, 2021 (Supplemental Digital Content File 1- Predefined Protocol, http://links.lww.com/MD/K319). This SRMA has been reported in accordance with the “Preferred Reporting Items for Systematic Reviews and Meta-analyses (PRISMA)” (Supplemental Digital Content File 2- PRISMA checklist, http://links.lww.com/MD/K320), and the “Meta-analysis of Observational Studies in Epidemiology (MOOSE)” guidelines (Supplemental Digital Content File 3- MOOSE Checklist, http://links.lww.com/MD/K321).^[22,23]^

### 2.1. Article selection and participants

We included randomized controlled trials (RCTs), observational studies, and cohort studies that used adjusted analyses to compare the outcomes of LE RUSS (≥1 screening) between adult patients with trauma (age >16 years) who underwent the assessment and those who did not undergo the assessment. We excluded case reports, conference abstracts or proceedings, duplicate publications, pediatric cohort studies, editorials, surveys, non-comparative studies, and studies involving patients with no trauma.

### 2.2. Interventions and controls

LE RUSS was performed at a certain frequency to facilitate the detection of DVT and prevent complications (such as phlegmasia, PE, and post-thrombotic syndrome) in adults at a high risk of VTE after a severe injury. The control group included patients who did not undergo RUSS; in such patients, the standard of care comprised a duplex US examination that was performed at the discretion of the treating physician if they suspected DVT.

### 2.3. Outcome measures

The outcomes were divided into primary/critical and secondary/non-critical outcomes. The critical outcomes comprised the rates of in-hospital and 90-day mortality; rates of in-hospital and 90-day proximal DVT (i.e., DVT above the knee) and distal DVT; rates of in-hospital and 90-day distal DVT progression; rates of in-hospital and 90-day PE; rates of fatal PE; and safety outcomes (Supplemental Digital Content Table 1- Outcome Definitions, http://links.lww.com/MD/K323). The non-critical outcomes comprised the time to VTE diagnosis (including times to distal DVT, proximal DVT, and PE diagnoses), durations of in-hospital stay and intensive-care-unit (ICU) stay, and ventilator days.

### 2.4. Search methods

We performed a comprehensive search of Medline, Embase, and the Cochrane Central Register of Controlled Trials databases from inception to October 18, 2021. A medical librarian was assigned to design the search strategy. Retrieval was limited to English literature. Search results were imported into a reference management software (EndNote version 20, New York), duplicates were removed, and imported into Covidence Melbourne, Australia.^[24]^ Duplicates between Medline and Embase records were removed using Ovid default duplicate detection, while any additional duplicates were removed using EndNote and Covidence. The keywords were chosen based on predefined terms and reviewing the Medical Subject Headings terms of articles. We included the following keyword search terms: VTE, PE, proximal DVT, distal DVT, RUSS, and trauma (Supplemental Digital Content File 4- Search Strategy, http://links.lww.com/MD/K322). Additionally, we manually searched all relevant narrative reviews’ reference lists in cases of pertinent papers.

### 2.5. Data abstraction

Two reviewers (A.A. and M.A.) screened all citations independently and in duplicate in 2 stages (first titles and abstracts followed by full texts) to identify eligible studies. A citation identified as potentially eligible by either reviewer in the first stage advanced to the second stage. In the second stage, disagreements were resolved by discussion or a third person (W.T.) to identify reasons for exclusion. Data extraction was performed by 2 reviewers (A.A. and M.A.) who used standardized forms for data extraction in Covidence (Supplemental Digital Content Table 2- Abstraction Sheet, http://links.lww.com/MD/K324). Any disagreement was analyzed and resolved by discussion, consensus, and when necessary, consultation with a third reviewer (W.T.). W.T. and K.M. identified any additional studies not identified by the search strategy. The purpose of data abstraction was to obtain the study population, injury types, US frequency, VTE prophylaxis types (mechanical and pharmacological), and outcome data.

### 2.6. Risk of bias assessment and grading of recommendations, assessment, development, and evaluations (GRADE) profile

We assessed the risk of bias (ROB) independently (A.A. and M.A.) and in duplicate for each study using a Cochrane tool for RCTs that classified ROB as “low,” “probably low,” “probably high,” or “high” for each of the following items^[25]^: randomization and sequence generation, concealment, blindness, analytical methods, allocation, incomplete data, selective outcome reporting, and other ROB. We assessed the ROB for observational studies using the risk of bias in non-randomized studies of interventions tool that classified ROB as “low risk,” “moderate risk,” “serious risk,” and “critical risk” for each of the following domains^[26]^: bias based on confounding, enrolling participants into the study, sorting interventions, deviations from intended interventions, incomplete data, measuring outcomes, and including reported result. Investigator with expertise in the grading of recommendations, assessment, development, and evaluations (GRADE) approach assessed the overall certainty in the pooled estimates for each outcome.^[27]^ Certainty assessment are based on several factors, including study design, ROB, consistency, directness, precision, and publication bias. In accordance with recommendations for interventional reviews, pooled data from RCTs started as high certainty and pooled data from observational studies as low certainty. Quality of evidence was categorized as high, moderate, low, and very low and we created GRADE evidence profile using GRADEpro Guideline Development Tool v3.6.1 (McMaster University and Evidence Prime, 2022). In cases of inconsistency between RCT results and observational data, we highlighted the findings with a higher certainty.

### 2.7. Statistical analysis

We performed the meta-analyses using the inverse variance weighting and Review Manager v5.3 (Cochrane Collaboration, Oxford, United Kingdom).^[28]^ We specified no prior subgroup analyses. Paralleling published guidance and due to methodological heterogeneity, RCTs and observational studies were pooled separately.^[29]^ Studies with sufficient clinical and methodological commonality were combined into meta-analyses. Forest plots were utilized to represent qualitative RCTs and quantitative synthesis of observational studies, displaying meta-analysis and analyzing the results in different subgroups among studies.^[30]^ We calculated relative risks (RRs) and mean differences (MDs) for dichotomous and continuous outcomes, respectively, with corresponding 95% confidence intervals (CIs). For continuous outcomes, we assumed a normal distribution (i.e., median = mean) and converted interquartile range to standard deviation using the methods suggested by the Cochrane handbook for systematic reviews of interventions and the methods described by Wan et al^[27,31]^ We considered both the random and fixed effects models considering study heterogeneity and the risk of small studies effects (fixed-effect model was considered when the number of studies was <3). Clinical and methodological heterogeneity across the studies were evaluated by examining the patients’ data, baseline data, interventions, and outcomes to determine sufficient similarities between studies. Additionally, we used the I^2^ statistics to assess the heterogeneity (I^2^ of 30%–60% and >60% as moderate and substantial heterogeneity, respectively), and chi squared test for homogeneity using visual inspection of forest plots. We considered the magnitude and direction of heterogeneity to rate down the certainty in evidence for inconsistencies. We performed sensitivity analysis for observational studies with low-to-moderate ROB (excluding high ROB studies). For each outcome, we performed additional sensitivity analysis for observational studies of adjusted odds ratios (ORs) and hazard ratios (HRs) using the random-effects method for estimation of between study variances. In this analysis, we excluded studies that did not provide ORs or HRs with corresponding CIs and which were adjusted for confounding by accounting for at least one of the following: type of pharmacological thromboprophylaxis received (unfractionated heparin/low-molecular-weight heparin), mechanical VTE prophylaxis use, injury severity score, abbreviated injury scale, acute physiology and chronic health evaluation (APACHE) II score, and the presence of femoral CVC at baseline. In accordance with Cochrane guidance, we combined studies with dichotomous outcome data (OR) and time-to-event data (HR) when the event rate was low (<10%).^[29]^ We present results as pooled adjusted OR (aORs) with 95% CIs (most conservative estimates that are available) as opposed to pooling events and totals.

## 3. Results

### 3.1. Search results and study characteristics

**Figure 1.**
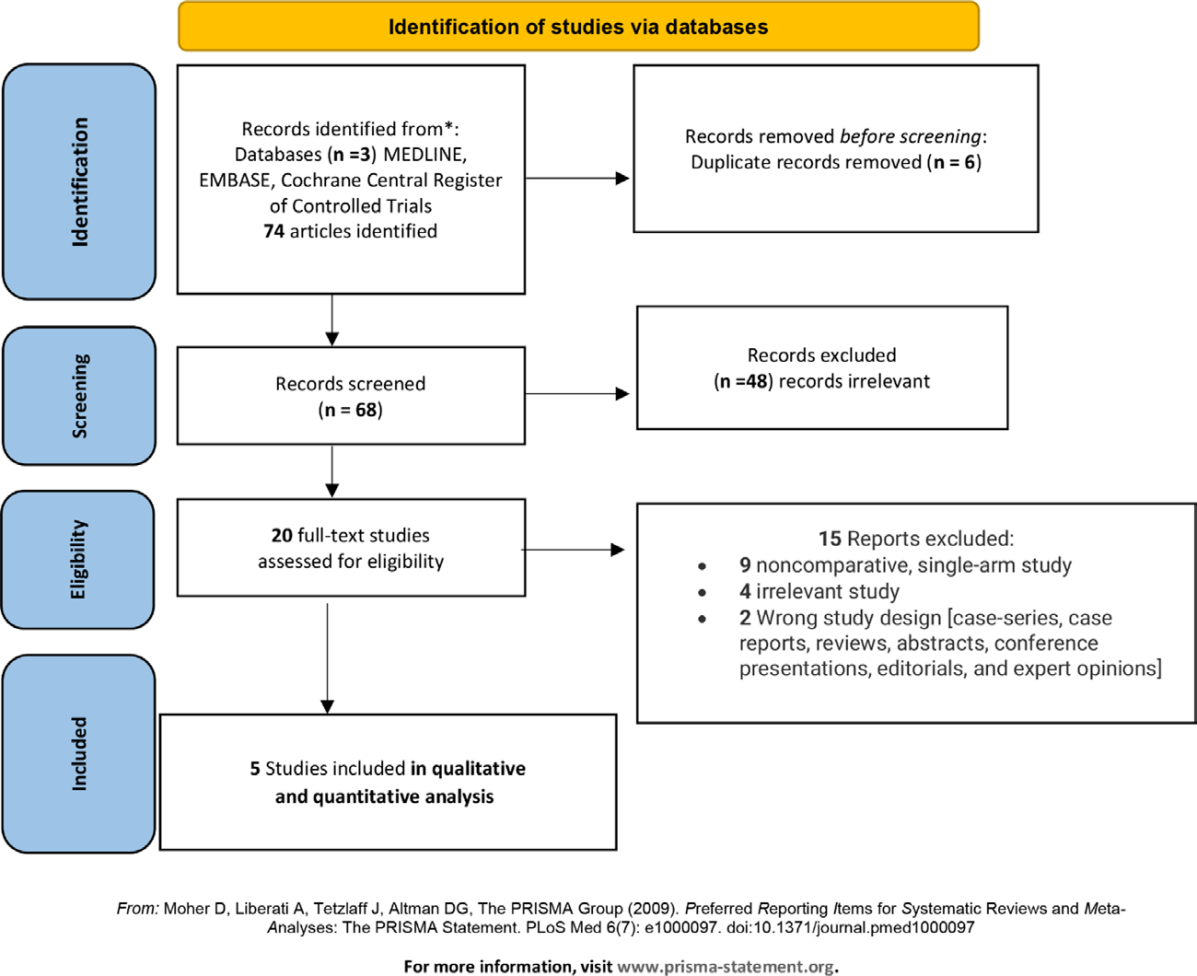
“Preferred Reporting Items for Systematic Reviews and Meta-Analyses” flow diagram depicting the updated search strategy.

Of the 1685 citations (Fig. 1) identified, we retrieved 20 full texts and reviewed 5 controlled studies (4 observational studies^[3–5,32]^ and an RCT^[33]^) (Table 1 and Supplemental Digital Content Table 3, http://links.lww.com/MD/K325- Details of the Trials). Of the observational studies, Arabi et al^[32]^ performed a pre- planned sub-study of the Prevention of Recurrent Venous Thromboembolism (PREVENT) trial and included patients with and without trauma.^[32]^ The included studies were predominantly large observational cohorts^[3–5,32]^ and an RCT^[33]^ (from the United States); an exception was the PREVENT multicenter study.^[32]^ Two studies^[3,33]^ included adults with trauma and moderate- or high-risk for VTE using a risk assessment profile (RAP) score ≥5 in a study^[33]^ or >10 in another study.^[3]^

**Table 1:** Characteristics of included studies.

Study	Country	Study design	Data source	Patient population	Sample size	Mean/median age (yr) ± SD	ISS ± SD	Screening US	Pharmacological VTE prophylaxis and mean initiation time	Mechanical VTE Prophylaxis
Kay et al (2021)^[33]^	USA	RCT	Single center	□ Adults w/t trauma (moderate- or high-risk defined by a RAP score of ≥5) □ 97% had blunt trauma	1989	62 ± 20.1	14 ± 9.7	□ RUSS group: serial LE US on 1^st^, 3^rd^, and 7^th^ in hospital days and thereafter weekly till discharge. □ No US for upper extremities and neck □ Non-RUSS group: at the discretion of trauma provider based on clinical suspicion of VTE	□ 75% LWMH □ 20% UFH □ 2%–3% DOACs □ MIT was about 23 h in both groups	NR
Allen CJ et al (2016)^[3]^	USA	Observational study	Single center	□ Asymptomatic trauma patients (as high risk for VTE RAP >10) □ 70%–80% had blunt trauma □ 40 % had TBI	402	47 ± 19	6.32 ± 1.87	□ RUSS group: Bilateral LE US weekly from the ankle to the inguinal ligaments. □ Proximal venous system (above or including the popliteal vein), Calf veins, Neck, and UE were not included. □ non-RUSS: If symptoms indicated by clinical suspicion of DVT	□ About 9%–10% did not receive any PVTEPx □ 90% received PVTEPx □ 40%–50% UFH □ 7%–16% Enox. □ MIT around 3^rd^ hospital day.	SCD was used if not prohibited by plaster immobilizer or external fixators
Haut et al (2007)^[4]^	USA	Observational study	Single center	□ Before and after study (i.e., 2 phases before (1995–1997), and after (1999–2005) □ RUSS group are less injured and less likely to have penetrating injuries	7559	□ 32.1 in the before phase patients □ 33.6 in the after-phase patients	□ 11.7 in the before phase patients □ 8.9 in the after-phase patients	□ RUSS group (after-phase patients), for asymptomatic trauma patients at high risk for DVT. □ non-RUSS (before-phase patients)	□ Enox. (dosed 30 mg twice daily). □ NR per group □ NR MIT	NR
Shackford et al (2016)^[5]^	USA	Observational study	Two centers	□ Comparison between 2 hospitals (i.e., Scripps Mercy hospital = RUSS group and Christiana Care hospital = non-RUSS group) w/t □ Overall VTE risk between the 2 groups based on ISS was similar. □ Included neurotrauma patients TBI and spinal cord injury	1226	□ 49.5 ± 22.4 (n = 772) Scripps Mercy □ 57.4 ± 23.7 (n = 454) Christiana Care	10 in both centers.	□ RUSS group (Scripps Mercy), LE US twice weekly for patients admitted to ICU, and weekly for patients admitted to the trauma floor □ non-RUSS (Christiana Care), LE US only for symptomatic patients	□ Significantly differ between groups □ 57% PVTEPx in RUSS □ 80% PVTEPx in non-RUSS □ PVTEPx held for 48 h in RUSS and for 72 h in non-RUSS patients w/t intracranial hemorrhage	□ Significantly differ between groups □ 94% used IPC in RUSS patients □ 60% used IPC in non-RUSS patients
Arabi et al (2020)^[32]^ Sub-study of PREVENT trial	Kingdom of Saudi Arabia	Observational study	Ten centers	□ From all PREVENT trial patients, only trauma patients included in this sub-study (8.5% RUSS group and 7.1% non-RUSS group) □ Downgraded for indirectness by 1 point	2065	□ 58.6 ± 20.6 RUSS group (N = 1682) □ Non-RUSS 54.1 ± 19.6 (N = 383)	NR	□ RUSS group, all proximal, distal, LE, UE, and neck US within 48 h then twice weekly □ Non-RUSS, requested based on clinical suspicion.	□ PVTEPx was equal between group □ 60%–65% UFH □ 35%–40% LMWH	□ 54.6% used IPC in RUSS group □ 30% used IPC in non-RUSS group

DOACs = direct oral anticoagulants, DVT = deep vein thrombosis, Enox = enoxaparin sodium, ICU = intensive-care-unit, IPC = intermittent pneumatic compression, ISS = injury severity score, LE = lower extremity, LWMH = low-molecular-weight heparin, MIT = mean initiation time, Non-RUSS = no routine US surveillance, NR = not reported, NR = not reported, PVTEPx = pharmacological VTE prophylaxis, RAP = risk assessment profile, RCT = randomized controlled trial, RUSS = routine US surveillance, SCD = sequential compression device, SD = standard deviation, TBI = traumatic brain injury, UE = upper extremity, UFH = unfractionated heparin, US = ultrasound, USA = United States of America, VTE = venous thromboembolism, w/t = with.

Majority of the studies predominantly included patients with blunt trauma as well as penetrating injuries, while 1 study included patients with traumatic brain and isolated spinal cord injuries.^[3]^ Five studies reported the use of pharmacological prophylaxis (low molecular weight heparin and unfractionated heparin in 5 and 4 studies, respectively). Pharmacological prophylaxis was typically initiated within 23 hours in an RCT^[33]^ and approximately 48 to 72 hours of admission in 3 observational studies^[3,5]^; and co-administration of mechanical prophylaxis in 3 other studies.^[3,5,32]^ The exclusion criteria common in the included studies were hypercoagulable state (e.g., presence of antiphospholipid syndrome, factor V Leiden, protein C and S deficiencies, and active cancer) and a history of VTE within 6 months. Importantly, distinction between proximal and distal LE DVTs as outcome measures was reported in 2 studies.^[32,33]^ RUSS screenings were reported with varying frequencies. In the RCT, the RUSS protocol during hospitalization included serial LE doppler US on the 1^st^, 3^rd^, and 7^th^ days followed by weekly RUSS until discharge.^[33]^ In the observational studies, 1 study^[3]^ performed RUSS weekly, while another^[5]^ performed RUSS twice weekly for patients in the ICU and once weekly for those admitted in the general ward. In the study by Arabi et al,^[32]^ RUSS was performed within the first 48 hours and twice weekly subsequently until discharge.^[32]^ The no-RUSS group was screened based on clinical suspicion. Another study^[4]^ did not specify the timing of RUSS in patients with asymptomatic trauma or the criteria in the no-RUSS group; however, the study specified that patients at high risk of DVT were screened.

### 3.2. Risk of bias

The Cochrane ROB Tool for RCTs revealed that the included RCT had a low ROB.^[33]^ The residual bias was attributed to the blinding domain of the participants, a lack of blinding of the clinicians responsible for ordering the serial US scans, and an unclear ROB in the blinding domain of the outcome assessors. The risk of bias in non-randomized studies of interventions tool revealed that 2 observational studies had a moderate ROB that was attributed to confounding arising from lack of adjustment for serious baseline differences. In one of these studies, most patients who underwent US surveillance required surgical interventions and sustained vascular and spinal cord injuries.^[3]^ In the other study, the pharmacological prophylaxis differed significantly among the participating centers.^[5]^ In 3 observational studies,^[3,5,32]^ bias was attributed to missing data, insufficient reporting of the doses administered for pharmacological prophylaxis, and differences in mechanical prophylactic co-interventions. (Supplemental Digital Content Fig. 1- Risk of Bias Assessment, http://links.lww.com/MD/K315).

### 3.3. Outcomes

**Figure 2.**
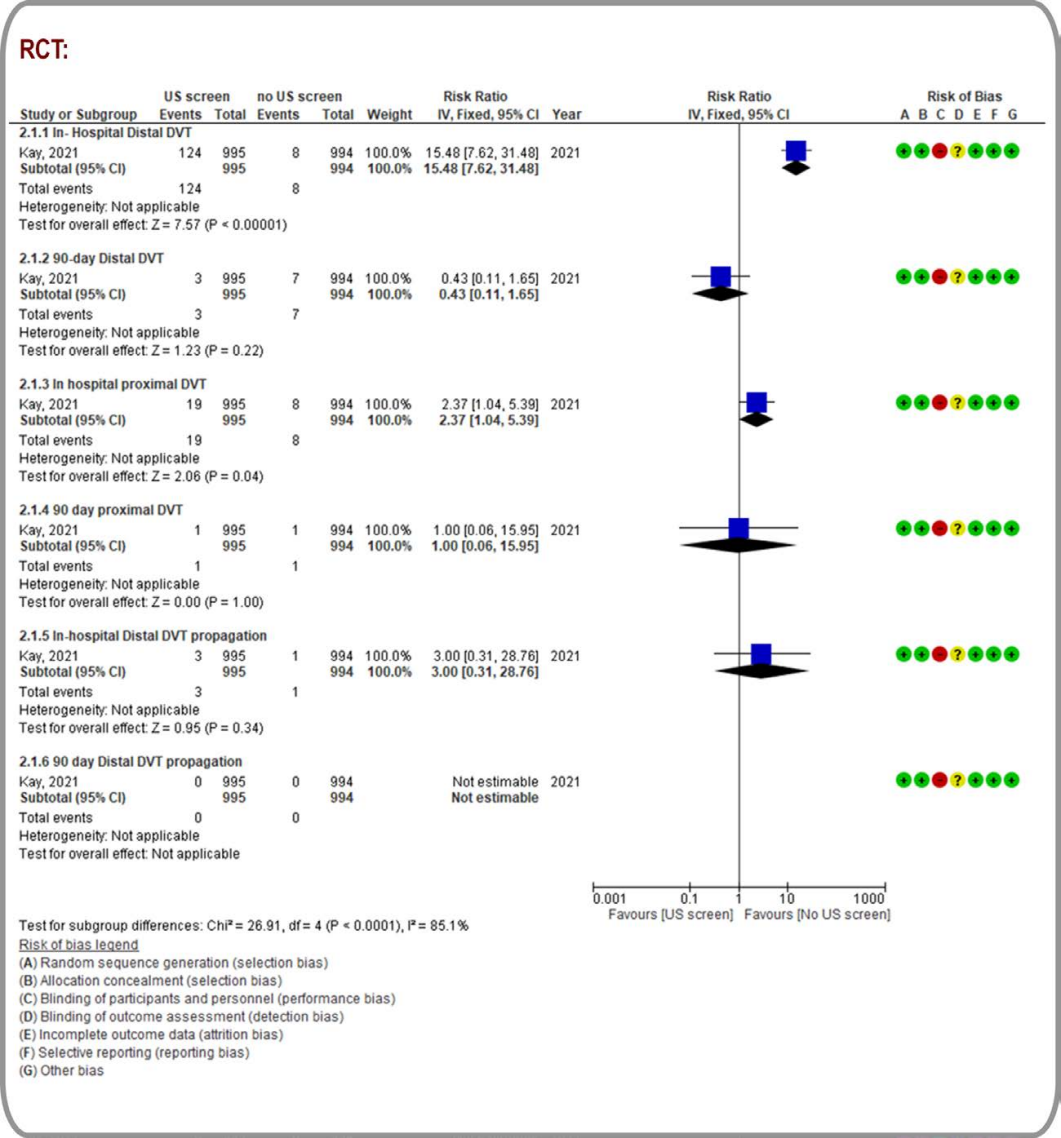
Proximal and distal deep vein thromboses: forest plot for randomized controlled trials.

**Figure 3.**
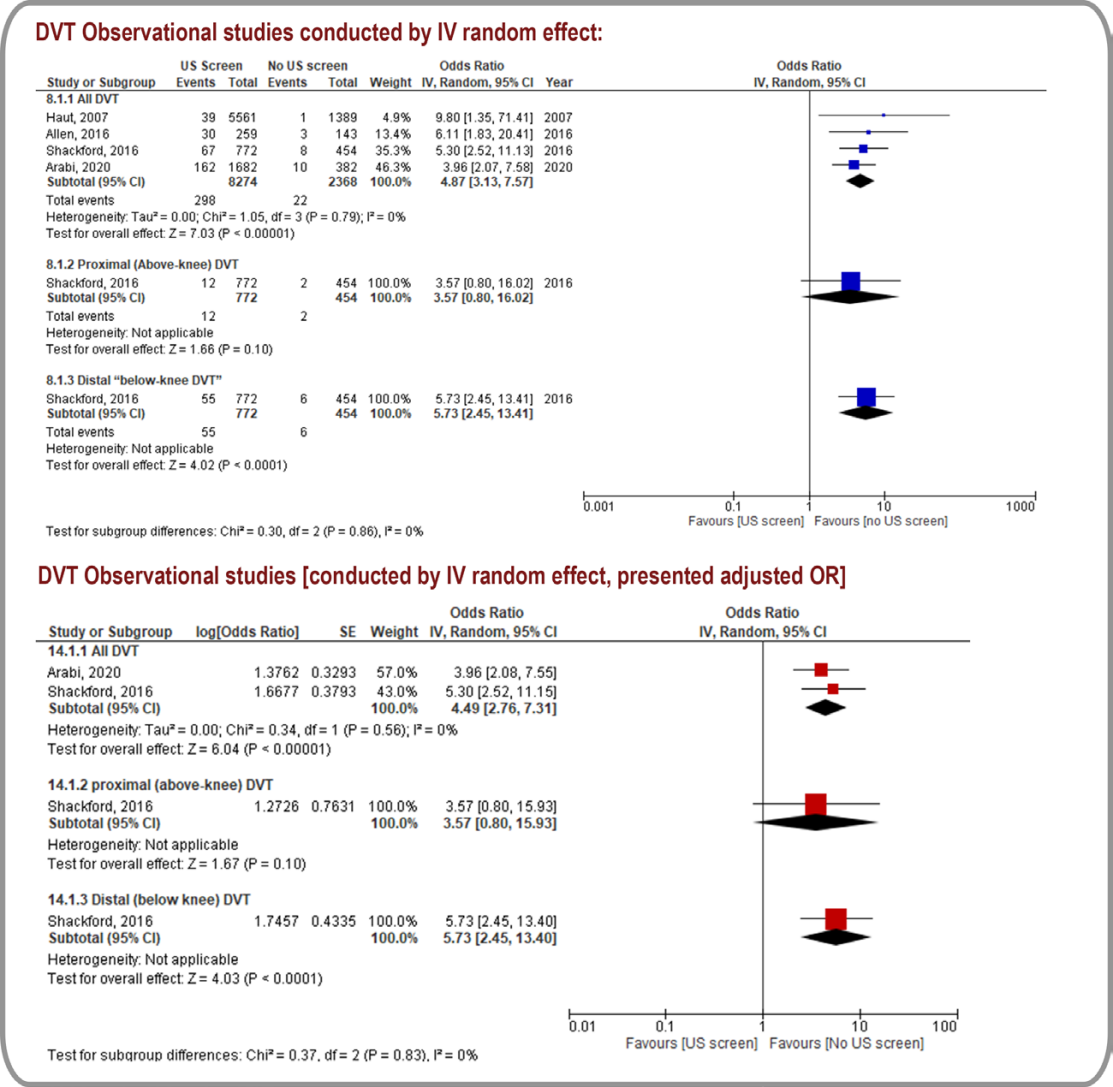
Proximal and distal deep vein thromboses: forest plot for observational studies.

**Figure 4.**
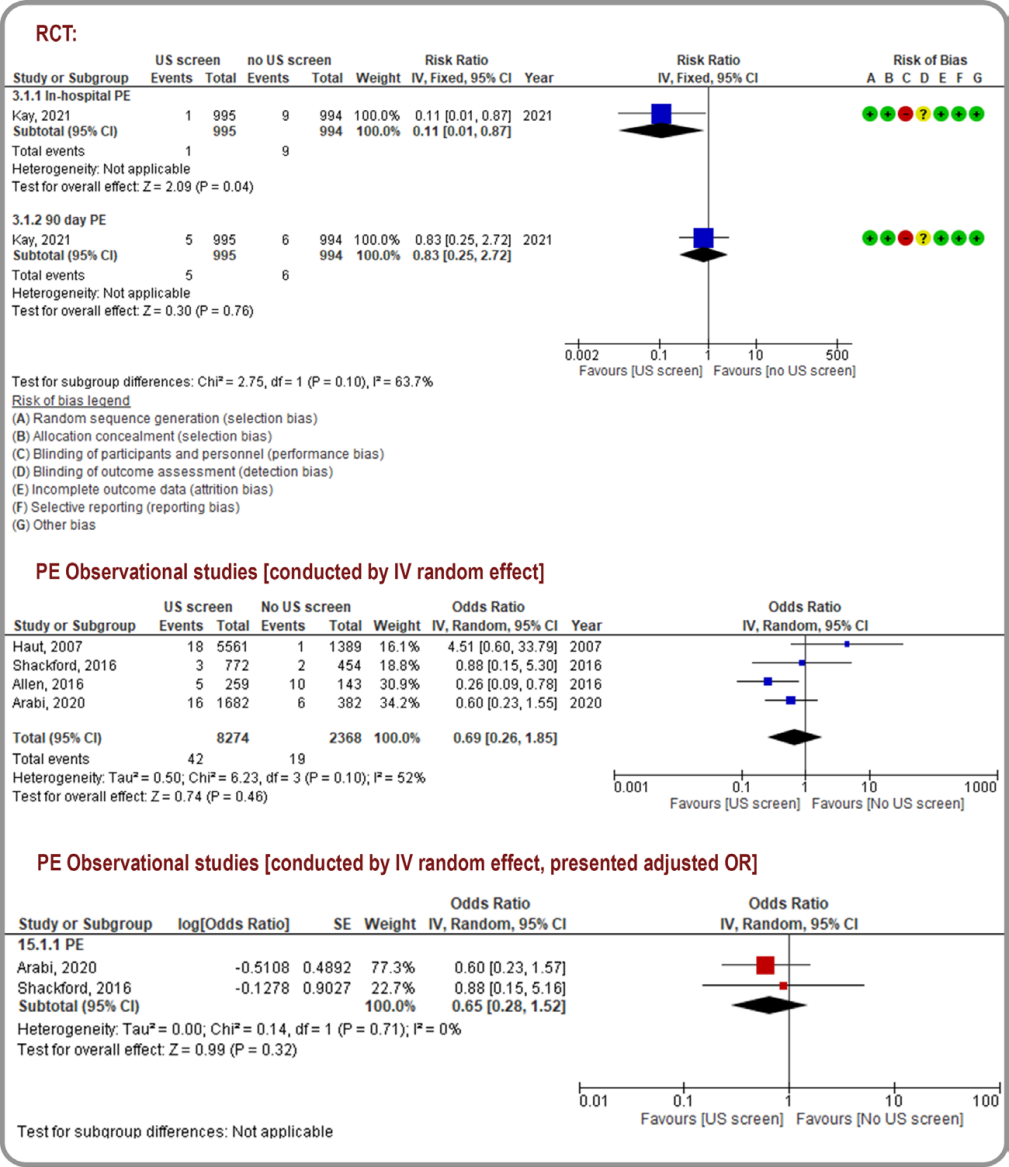
Pulmonary embolism: forest plot for randomized controlled trials and observational studies.

**Figure 5.**
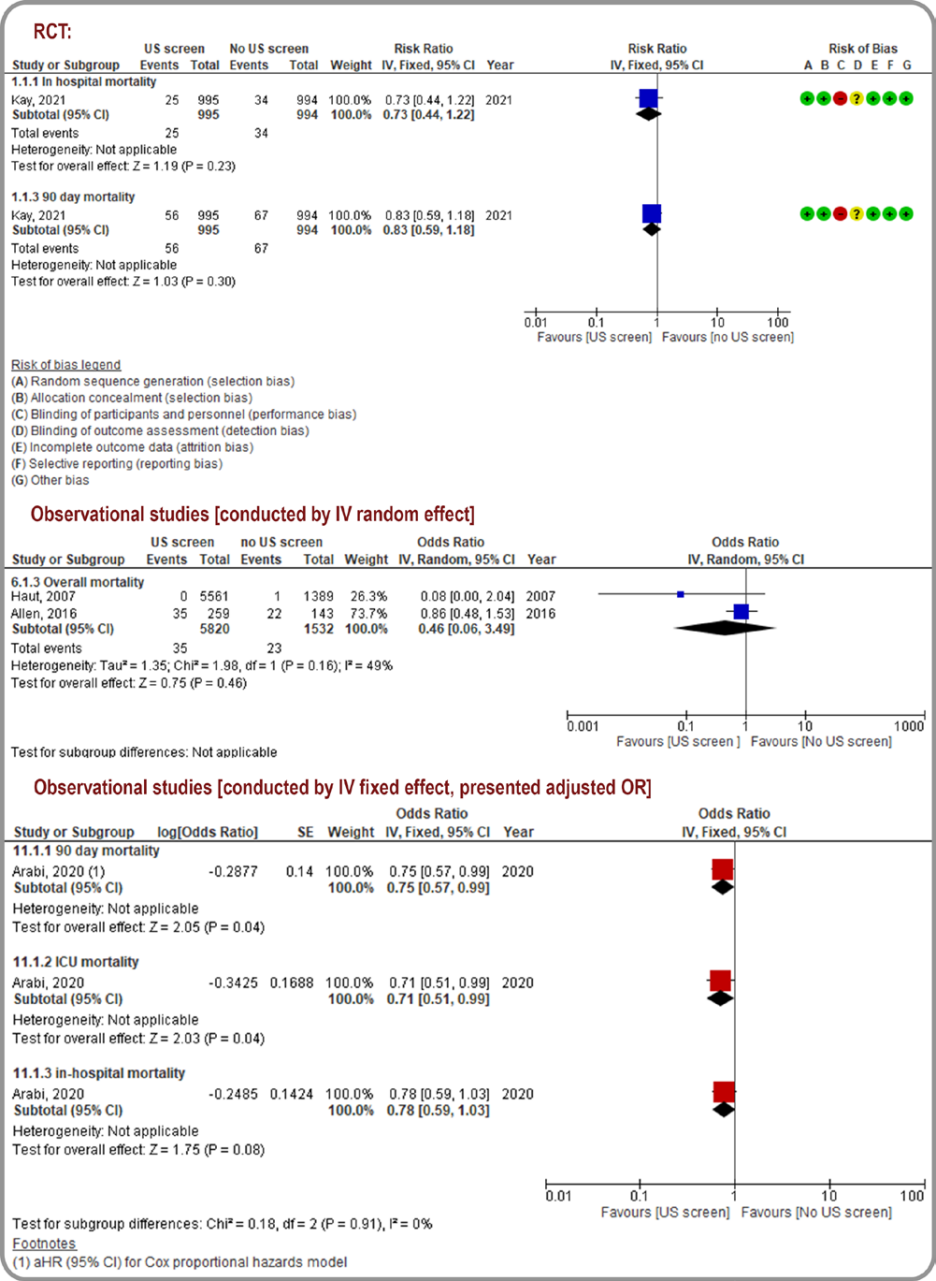
Mortality: forest plot for randomized controlled trials and observational studies.

Figures 2 to 5 and Table 2 present the outcomes in the form of forest plots (qualitative pooled data from RCTs and quantitative synthesis of pooled data from observational studies), and GRADE assessments, respectively. (Supplemental Digital Content Table 4- GRADE Certainty Assessments, http://links.lww.com/MD/K326) Further details are presented in (Supplemental Digital Content Fig. 2- Sensitivity Analysis, http://links.lww.com/MD/K316).

**Table 2:** Outcome GRADE assessments.

Outcome GRADE	Studies (N)	Pooled RR or OR (95% CI)	*P* value	I^2^ value	Certainty
In-Hospital distal DVT- RCT	1 RCT^[33]^	□ RR 15.48 (7.62–31.48)	Non-applicable	Non-applicable	⨁⨁◯◯ Low
In-Hospital proximal DVT-RCT	1 RCT^[33]^	□ RR 2.37 (1.04–5.39)	Non-applicable	Non-applicable	⨁◯◯◯ Very low
In-hospital, Distal DVT propagation-RCT	1 RCT^[33]^	□ RR 3.00 (0.31–28.76)	Non-applicable	Non-applicable	⨁◯◯◯ Very low
All DVT- observational	4 studies^[3–5,32]^	□ OR 4.87 (3.13–7.57)	*P* < .00001	I^2^ = 0%	⨁◯◯◯ Very low
All DVT- observational adjusted OR	2 Observational study^[5,32]^	□ Adjusted OR 4.49 (2.76–7.31)	*P* < .00001	I^2^ = 0%	⨁◯◯◯ Very low
In-hospital PE -RCT	1 RCT^[33]^	□ RR 0.11 (0.01–0.87)	Non-applicable	Non-applicable	⨁◯◯◯ Very low
PE observational studies	4 Observational studies	□ OR 0.69 (0.26–1.85)	*P* = .46	I^2^ = 52%	⨁◯◯◯ Very low
PE- observational adjusted OR	2 Observational study^[5,32]^	□ Adjusted OR 0.65 (0.28–1.52)	*P* = .32	I^2^ = 0%	⨁◯◯◯ Very low
Time to distal DVT Dx -RCT	1 RCT^[33]^	□ MD 1.55 lower (4.22 lower to 1.12 higher)	Non-applicable	Non-applicable	⨁◯◯◯ Very low
Time to proximal DVT Dx -RCT	1 RCT^[33]^	□ MD 2.25 lower (5.74 lower to 1.24 higher)	Non-applicable	Non-applicable	⨁◯◯◯ Very low
90-d mortality-RCT	1 RCT^[33]^	□ RR 0.83 (0.59–1.18)	Non-applicable	Non-applicable	⨁⨁◯◯ Low
In-hospital mortality-RCT	1 RCT^[33]^	□ RR 0.73 (0.44–1.22)	Non-applicable	Non-applicable	⨁◯◯◯ Very low
90-d mortality (PREVENT sub-study)*	1 Observational study^[32]^	□ HR 0.75 (0.57–0.99)	Non-applicable	Non-applicable	⨁◯◯◯ Very low
ICU mortality (PREVENT sub-study)*	1 Observational study^[32]^	□ HR 0.71 (0.51–0.99)	Non-applicable	Non-applicable	⨁◯◯◯ Very low
In hospital mortality (PREVENT sub-study)*	1 Observational study^[32]^	□ HR 0.78 (0.59–1.02)	Non-applicable	Non-applicable	⨁◯◯◯ Very low

DVT = deep vein thrombosis, GRADE = grading of recommendations, assessment, development, and evaluations, HR = hazard ratio, I^2^ value = test of heterogeneity, ICU = intensive-care-unit, MD = mean differences, OR = odds ratio, PE = pulmonary embolism, *P* value = probability value, RCT = randomized controlled trial, RR = relative risk.

* Assessed with: Cox proportional hazards model.

### 3.4. DVT

The RUSS group in the included RCT (N = 1989) was associated with increased rates of in-hospital distal DVT (RR, 15.48; 95% CI, 7.62 − 31.48; low certainty) and in-hospital proximal DVT (RR, 2.37; 95% CI, 1.04 − 5.39; very low certainty). However, there were no significant differences in the rates of 90-day proximal and distal DVTs between the RUSS and no-RUSS groups.^[33]^ The likelihood of in-hospital distal DVT progression was higher in the RUSS group than in the no-RUSS group (RR, 3; 95% CI, 0.31–28.76; very low certainty; Fig. 2). Evidence from observational studies (four studies, N = 10,642) suggested higher odds of DVT detection using RUSS (OR, 4.87; 95% CI, 3.13–7.57; very low certainty).^[3–5,33]^ Similarly, pooled estimates of aORs from 2 observational studies also revealed that RUSS was potentially associated with higher odds of DVT detection (aOR, 4.49; 95% CI, 2.76–7.31; very low certainty; Fig. 3).^[5,32]^ After excluding a study with a high ROB,^[4]^ sensitivity analyses of studies with a low-to-moderate ROB revealed similar trends (OR, 4.69; 95% CI, 2.98–7.38; Supplemental Digital Content Fig. 2- Sensitivity Analysis, http://links.lww.com/MD/K316).

### 3.5. Pulmonary embolism (PE)

PE events were grouped by detection time (in the hospital or within 90 days of follow-up). Kay et al^[33]^ demonstrated a significantly lower rate of in-hospital PE detection in the RUSS group than in the no-RUSS group (RR, 0.11; 95% CI, 0.01–0.87; very low certainty, Fig. 4).^[33]^ After excluding the high ROB study,^[4]^ sensitivity analysis of 3 observational studies with a low-to-moderate ROB (N = 3692) reduced heterogeneity and revealed a reduction in the PE detection rates with RUSS (OR, 0.47; 95% CI, 0.24–0.9; I^2^ = 0; very low certainty).^[3,5,33]^ Furthermore, pooled estimates of aORs from 2 observational studies revealed that RUSS was potentially associated with lower odds of PE detection (aOR, 0.65; 95% CI, 0.28–1.52; very low certainty); however, the 95% CI could not exclude an absence of differences or a clinically important benefit. These results are limited by indirectness and serious imprecision.^[5,32]^

### 3.6. Mortality

In the included RCT, no difference was observed between the in-hospital mortality (RR, 0.73; 95% CI, 0.44–1.22; very low certainty) and 90-day mortality (RR, 0.83; 95% CI, 0.59–1.18; low certainty) in both the RUSS and no-RUSS groups. Similarly, the effects of RUSS on mortality were uncertain in 2 observational studies (pooled OR, 0.46; 95% CI, 0.06–3.49; very low certainty).^[3,4]^ After excluding the high ROB study,^[4]^ sensitivity analysis of studies with a low-to-moderate ROB revealed identical trends in the invariable results. In one observational study, analysis using a Cox proportional hazards model revealed that compared with the no-RUSS group, the RUSS group was associated with lower 90-day adjusted mortality and 90-day adjusted ICU mortality (HR, 0.51; 95% CI, 0.51–0.99; very low certainty); however, no intergroup differences were observed in the in-hospital mortality (HR, 0.78; 95% CI, 0.59–1.02; very low certainty; Fig. 5).^[32]^ (Supplemental Digital Content Fig. 3- Sensitivity analysis for low-moderate ROB studies, http://links.lww.com/MD/K317).

### 3.7. Time to VTE diagnosis

In the included RCT, times to both proximal DVT diagnosis (MD, 2.25 days shorter; 95% CI, 5.74–1.24; very low certainty) and distal DVT diagnosis (MD, 1.56 days shorter; 95% CI, 4.22–1.12; very low certainty) were shorter in the RUSS group than in the no-RUSS group. Furthermore, the average time to proximal DVT development was 90 days in the RUSS group and 8 days in the no-RUSS group.^[33]^ The average time to distal DVT development was 124 days in the RUSS group and 8 days in the no-RUSS group. One observational study reported on the time of VTE diagnosis and revealed that RUSS was associated with an earlier diagnosis of DVT (MD, 14 days shorter; 95% CI, 14.53–13.47) and PE (MD, 10 days shorter; 95% CI, 12.04–8.64).

### 3.8. Bleeding outcomes associated with unnecessary administration of therapeutic doses of anticoagulants in the treatment of clinically silent DVT

For distal DVT, 2 of 8 patients (25.0%) in the no-RUSS group and 11 of 124 patients (8.9%) in the RUSS group received full doses of anticoagulants (*P* = .18). For proximal DVT, 4 of 8 patients (50.0%) in the no-RUSS group and 14 of 19 patients (73.7%) in the RUSS group received full doses of anticoagulants (*P* = .38). No intergroup differences were observed in the major bleeding rate and no association of increased bleeding risk was observed with RUSS group (RR, 1.24; 95% CI, 0.31–4.92; (Supplemental Digital Content Fig. 4, http://links.lww.com/MD/K318- Forest plot for major bleeding).^[33]^

## 4. Discussion

### 4.1. Main findings

This SRMA summarized the best available evidence on the role of RUSS in adult patients with trauma and its effects on the outcomes of this population. RUSS may result in improved DVT detection rates, fewer cases of PE, shorter times to DVT diagnosis, and uncertain effects on mortality. Pending further information, very-low-certainty data support the use of routine bilateral LE US for screening asymptomatic DVT in adults with trauma who are at a high risk of VTE but are not candidates for pharmacological VTE prophylaxis.^[9,10,14–16]^ A possible explanation for this is that the diagnosis and treatment of DVT reduced the following: need for additional investigations for PE, progression of proximal DVT, and embolization of clots (thereby preventing fatal PE). Early detection and diagnosis will enable initiation of appropriate therapies, such as systemic anticoagulants or inferior vena cava filter placement in patients whose conditions are contraindicated for systemic anticoagulants. Concordantly, our results agree with these reports and support using RUSS to diagnose silent DVT in adults with trauma.

Diagnostic studies have revealed that LE DVT has an estimated incidence of 10 to 90% in patients with head injuries.^[34,35]^ Most patients (86%) with trauma who underwent RUSS weekly were diagnosed with LE DVT but were clinically asymptomatic; this highlights the silent nature of LE DVT.^[36]^ Therefore, implementing RUSS can lower the incidence rates of PE and the subsequent morbidity in and mortality of seriously injured patients.^[3]^ Furthermore, Kadyan et al^[37]^ and Bandle et al^[12]^ reported that unlike in patients with a low-to-moderate risk of DVT, RUSS is both lifesaving and cost-effective in patients with a spinal cord injury and in those who are at a high risk of DVT after trauma.^[12,37]^ Contrastingly, Cipolle et al^[2]^ failed to identify an association between RUSS and a reduction in the incidence of PE. They concluded that the PE incidence was more influenced by the patient compliance with the pharmacological VTE prophylaxis protocol (i.e., administration of un-fractionated subcutaneous heparin followed by conversion to low-dose warfarin) than by RUSS. The authors emphasized that adherence to a prophylactic pharmacological protocol was more crucial than RUSS for preventing VTE sequelae.

Existing literature on the role of RUSS in patients with trauma remains controversial owing to non-uniform findings regarding the clinical application of RUSS and concerns of surveillance bias.^[3,15,17,19]^ Some healthcare agencies have linked the incidence of in-hospital VTE with the quality of medical care provided and have proposed that hospitals with higher rates of VTE should face punitive diminutions in reimbursements.^[4]^ Huat et al^[4]^ implemented a more aggressive RUSS protocol for DVT in patients with trauma and reported a 10-fold increase in the DVT detection rate, thereby demonstrating a significant surveillance bias. Pierce et al^[38]^ analyzed data from the National Trauma Data Bank and identified a similar surveillance bias in hospitals across the United States. Furthermore, a survey of 317 surgeons revealed wide variations in the individual opinions on the need for DVT screening among asymptomatic patients with trauma; 53%, 36%, and 11% agreed, disagreed, and neither agreed nor disagreed with the screening, respectively. Furthermore, approximately 73% of the responders in this survey thought that patients should be screened early (i.e., within the first 6 days of admission). Moreover, approximately 61% of the respondents thought that a duplex US examination should be performed every week. Approximately 60% of the respondents worked at a trauma center that did not follow published guidelines or protocols for duplex US-based screening for DVT among asymptomatic patients with trauma.^[2]^

The clinical efficacy of RUSS for post-trauma VTE prophylaxis is still debated. Some guidelines recommend RUSS to detect asymptomatic DVT in trauma patients at high risk of VTE, but they do not agree on the associated risk or the effect of RUSS on PE rates.^[39–41]^ They even discourage reporting the rate of DVT as a hospital outcome since RUSS would essentially elevate it.^[40]^ In patients with low-risk of DVT, many studies argue that RUSS does not decrease the rate of PE or fatal PE, may cause false positives, and is costly for this population.^[40,42]^ Although most studies use the Greenfield RAP score >10 to identify risk of VTE and thereby, select patients for RUSS, others employ the abbreviated injury scale score >3 as a risk factor for VTE in patients with trauma.^[12,40,42]^ A brief overview of current opinion of major surgical societies in the management of trauma-related VTE can be found in (Supplemental Digital Content Table 5, http://links.lww.com/MD/K327- Recommendations from major societies on post-trauma thromboprophylaxis).

Previous cost-effective analyses have focused on the financial costs of patient care.^[2,9]^ Studies that oppose RUSS have suggested that its associated costs are prohibitive,^[8]^ although its selective use for high-risk patients is beneficial.^[12]^ The expected healthcare costs at 12 weeks for patients admitted to Canadian ICUs for severe injuries and believed to have contraindications for pharmacological prophylaxis for up to 2 weeks because of a major bleeding risk were as follows: Can$55,831 for pneumatic compression device placement, Can$55,334 for RUSS, and Can$57,377 for inferior vena cava filter placement. Compared with the other 2 modalities, RUSS was associated with better clinical outcomes and lower costs. Lifetime analysis revealed that the expected quality-adjusted life years were similar for all 3 treatment modalities, although the costs remained the highest for inferior vena cava filter placement. In the base case analysis, RUSS was dominant over other modalities, as it was associated with better clinical outcomes and lower costs (both at 12 weeks and over a lifetime).^[43]^

This SRMA has several strengths. It involved a comprehensive search, adhered to the recommendations for meta-analysis of interventional studies, and used GRADE to assess the certainty in estimates.^[27]^ We considered obtaining evidence-based studies that utilized influential factors, such as main outcomes, chemoprophylaxis, and other mechanical prophylactic devices.

This review also has certain limitations, and its findings should be interpreted with caution. First, the study was based on aggregate published data, and adjustments for patient-level characteristics could not be performed. Second, significant clinical heterogeneity was observed among studies owing to differences in the RUSS frequency. Four of the 5 studies included in our review incorporated a screening test performed at least once in the first week, followed by tests performed weekly thereafter. Furthermore, 2 of these studies recommended RUSS twice a week.^[5,32]^ Additionally, Fraser et al^[44]^ suggested a week-long interval between 2 negative screenings to exclude DVT.^[44]^ We found that the assessment of DVT varied across the included studies; this heterogeneity was primarily attributed to the differing anatomical locations of DVT among these studies.

Third, the effect estimates of observational studies should be interpreted with caution owing to possible observer bias and residual confounders. Moreover, findings from the included observational studies were limited by indirectness.^[45]^ It important to mention that the GRADE methodology acknowledges the concept of “indirectness” and offers guidance on assessing the quality of evidence in these cases. Guyatt et al (2011)^[45]^ elaborated upon this aspect of GRADE guidelines and emphasized the significance of downgrading the quality of evidence when dealing with indirect evidence. Our SRMA has closely followed these principles. The population included in the PREVENT sub-study was a mix of medically, surgically, and critically ill trauma patients; in fact, patients with trauma accounted for 8% of all the included patients.

### 4.2. Future research

RCTs with adjusted or adaptive methodological study designs that incorporate Bayesian statistical methods^[46]^ are needed to compare the efficacies of RUSS, prophylactic inferior vena cava filter placement, and a combination of the 2 for preventing fatal PE as a clinically important VTE outcome. Further research is also required to define the optimal RUSS frequency. Although most included studies used the RAP score to identify asymptomatic patients with trauma who were at a high risk of VTE, further analysis is required to identify the optimal clinical VTE risk assessment method for determining populations that will benefit the most from RUSS. More studies are needed to confirm the utility and efficiency of RUSS for DVT in the upper extremities and neck. The “Diagnosing Deep-vein Thrombosis Early in Critically Ill Patients” trial is an ongoing RCT that is expected to provide valuable information on the comparative outcomes of RUSS and a clinician-directed approach for patients at a high risk of DVT admitted to the ICU (Y.M. Arabi, MD, unpublished data, ongoing, ClinicalTrials.gov Identifier: NCT05112705).

### 4.3. Conclusion

Our findings represent the latest and most comprehensive summary of RUSS outcomes of adults with trauma at a high risk of DVT. In adults with trauma who are not eligible for VTE prophylaxis and at a high risk of VTE, RUSS may result in better DVT detection rates, a lower incidence of PE, shorter times to DVT diagnosis, and uncertain effects on mortality. RUSS is noninvasive and widely available with little effects on equity, acceptability, cost-effectiveness, and feasibility. However, due to low detection rates of clinically relevant VTE and possible high costs, it may not be indicated for ambulatory patients with trauma or for those at a low risk of VTE. Pending further data, the very-low-certainty data in our SRMA support the use of routine bilateral LE US for screening asymptomatic DVT in adults with trauma who are at a high risk of VTE.

## Acknowledgments

We thank Kaitryn Campbell (MSc, MLS; St. Joseph Healthcare Hamilton, ON) for peer review of the MEDLINE search strategy.

## Author contribution

**Conceptualization:** Abdulaziz M. Al-Sharydah, Khalid Maghrabi, Wail Tashkandi, Marwa Amer.

**Data curation:** Abdulaziz M. Al-Sharydah, Khalid Maghrabi, Wail Tashkandi, Marwa Amer.

**Formal analysis:** Abdulaziz M. Al-Sharydah, Mohammed S. Alshahrani, Khalid Maghrabi, Wail Tashkandi, Marwa Amer.

**Investigation:** Abdulaziz M. Al-Sharydah, Mohammed S. Alshahrani, Khalid Maghrabi, Wail Tashkandi, Marwa Amer.

**Methodology:** Abdulaziz M. Al-Sharydah, Mohammed S. Alshahrani, Marwa Amer.

**Project administration:** Abdulaziz M. Al-Sharydah, Mohammed S. Alshahrani, Khalid Maghrabi, Wail Tashkandi, Marwa Amer.

**Resources:** Abdulaziz M. Al-Sharydah, Mohammed S. Alshahrani, Khalid Maghrabi, Marwa Amer

**Software:** Abdulaziz M. Al-Sharydah, Marwa Amer.

**Supervision:** Abdulaziz M. Al-Sharydah, Mohammed S. Alshahrani, Khalid Maghrabi, Wail Tashkandi, Marwa Amer.

**Validation:** Abdulaziz M. Al-Sharydah, Mohammed S. Alshahrani, Khalid Maghrabi, Wail Tashkandi, Marwa Amer.

**Visualization:** Abdulaziz M. Al-Sharydah, Mohammed S. Alshahrani, Marwa Amer.

**Writing – original draft:** Abdulaziz M. Al-Sharydah, Mohammed S. Alshahrani, Khalid Maghrabi, Wail Tashkandi, Marwa Amer.

**Writing – review & editing:** Abdulaziz M. Al-Sharydah, Mohammed S. Alshahrani, Khalid Maghrabi, Wail Tashkandi, Marwa Amer.

## Supplementary Material


























