# Supplementary material for: Ultrasound surveillance for deep venous thrombosis and subsequent venous thromboembolism in adults with trauma: A systematic review and meta-analysis
Source: Medicine (Baltimore). 2023 Oct 27;102(43):e35625. doi: 10.1097/MD.0000000000035625 (PMC10615543; doi:10.1097/MD.0000000000035625)
Supplement: Supplementary file 1 [file medi-102-e35625-s001.docx]

**Supplemental Digital Content File 1:**

**Predefined protocol designed on Oct 27, 2021.**

TITLE OF REVIEW: ULTRASOUND SURVEILLANCE FOR DEEP VENOUS THROMBOSIS AND SUBSEQUENT VENOUS THROMBOEMBOLISM IN ADULTS WITH TRAUMA: A SYSTEMATIC REVIEW AND META-ANALYSIS

CONFLICT OF INTEREST: None

QUESTION OF INTEREST: In adults with trauma who are not candidates for pharmacologic VTE prophylaxis, should we recommend routine VTE US screening versus no routine screening?

BACKGROUND: The practice of routine VTE ultrasound (US) scanning significantly varies between trauma centers. This disagreement also presents in high-risk symptomatic patients versus silent asymptomatic ones. This systematic review and meta-analysis will compare the use of routine VTE US screening versus no routine US screening in adults with trauma who are at high-risk of VTE.

METHODS: We will conduct this systematic review and meta-analysis by following the recommendations from the Preferred Reporting Items for Systematic Reviews and Meta-analyses guidelines.

Data Sources and Searches: We will perform a comprehensive search of MEDLINE, EMBASE and the Cochrane trial registry from inception. We will restrict our search to English language and human studies. An experienced health science librarian will assist in developing the search strategy. Keyword search terms will include: VTE, PE, proximal DVT, distal DVT, RUS, and trauma

Study Selection: Search results will be imported into reference management software (EndNote version 20), deduplicated, and imported into Covidence to facilitate the systematic review process. Two reviewers (AA, MA) will screen all citations independently and in in duplicate in two stages, first titles and abstracts, then full texts to identify eligible studies. A citation identified as potentially eligible by either reviewer at the first stage will be advanced to the second stage. In the second stage, disagreements will be resolved by discussion or third person (WT) adjudication if necessary. Reasons for exclusion at the second stage will be stated. We will include randomized clinical trial, cohort observational studies with adjusted analysis that examined the routine serial surveillance ultrasonography in adults ≥16 years with trauma, compared with control (standard of care- duplex ultrasounds obtained as indicated by physical examination when there a clinical suspicion of DVT at the discretion of the treating team), and reported any of the following critical outcomes: PE, fatal PE, deep vein thrombosis, venous thromboembolism, mortality, and/or adverse effects (bleeding associated with unnecessary use of full anticoagulation for asymptomatic DVT)

We will exclude duplicates, pediatric population < 16 years, conference abstract, editorials, surveys, or non-comparative studies.

Data Extraction and Quality Assessment :

Using a predefined data abstraction form, two reviewers (AA and MA) will complete data extraction independently and in duplicate. A third reviewer (WT) will resolve disagreements if necessary. The following will be abstracted: study characteristics, demographic data, type of screening US and frequency, co-interventions details (pharmacological and mechanical prophylaxis) and outcome data. We will contact individual study authors in cases of missing study date. Risk of bias will be assessed independently by 2 reviewers (AA and MA) and in duplicate for each study using a Cochrane risk of bias tool for RCT that classifies risk of bias as: “low”, “probably low”, “probably high” or “high” for each of the following items: randomization and sequence generation, allocation sequence concealment, blinding, incomplete data, selective outcome reporting and other risk of bias. We will evaluate the overall risk of bias as the highest risk attributed to any criterion. The Risk Of Bias In Non-randomized Studies - of Interventions (ROBINS-I) tool will be used to used to assess risk of bias in observational studies

Data Synthesis and Analysis:

We will perform all analyses using RevMan 5.3 (Cochrane Collaboration, Oxford) software. We will use inverse variance weighting. We will consider both random effects and fixed effects models considering study heterogeneity and the risk of small studies effects (fixed-effect model only when the number of studies was < 3). Results will be presented as relative risks or odds ratio for dichotomous outcomes and as mean differences for continuous outcomes, both with 95% confidence intervals. For continuous outcomes, we will assume a normal distribution and we will convert inter-quartile range to standard deviation using the methods suggested by Cochrane handbook for systematic reviews of interventions. We will assess for publication bias if the included RCTs are equal to or more than 10 studies. Heterogeneity will be assessed between studies using the χ 2 tests for homogeneity, the I2 statistic and the visual inspection of the forest plots. The magnitude and direction of heterogeneity will be considered when deciding whether to rate down our certainty in the evidence for inconsistency. Based on the characteristics of the included studies, a decision will be made regarding the need for sensitivity analysis.

Assessment of Certainty of Evidence: We will appraise the overall certainty of evidence for each outcome using the Grading of Recommendations Assessment, Development and Evaluation approach (Schünemann, #50;Schünemann, 2019 #50)
