# Supplementary material for: Ultrasound surveillance for deep venous thrombosis and subsequent venous thromboembolism in adults with trauma: A systematic review and meta-analysis
Source: Medicine (Baltimore). 2023 Oct 27;102(43):e35625. doi: 10.1097/MD.0000000000035625 (PMC10615543; doi:10.1097/MD.0000000000035625)
Supplement: Supplementary file 4 [file medi-102-e35625-s004.docx]

**Supplemental Digital Content Table 1: Outcome definitions used for the studies included**

| **Study** | **Definition** | **Duration of Follow-Up** |
| --- | --- | --- |
| Kay 2021 (1) | **DVT**   - the providers performed daily history and physical examinations targeted at evaluating for signs and symptoms of DVT, guided by the Wells criteria. - Symptomatic DVT in both groups was pursued with a diagnostic DUS scan. Any in-hospital DVT identified in the No Us group or in either group at 90 days was considered symptomatic. Deep venous thrombosis diagnosis was characterized as either below-knee, defined as thrombus confined to the calf venous system, or above-knee, defined as thrombus above or including the popliteal vein   **PE**   - Symptoms warranting investigation for PE included tachycardia, hypoxia, oxygen desaturation, dyspnea, fever, or hypotension and was worked up with a computed tomographic pulmonary angiogram (CTPA) at the discretion of the trauma provider   **Deep venous thrombosis propagation**   - defined as extension of a known below-knee clot to the popliteal vein or above. Asymptomatic DVT in the US group was diagnosed on the day of the scheduled DUS, generally within a few hours upon completion of the study.   **Major bleeding**   - determined using the criteria published by the International Society on Thrombosis and Hemostasis and included fatal bleeding; symptomatic bleeding in a critical area or organ such as intracranial, intra-abdominal, or retroperitoneal; and bleeding resulting in a hemoglobin drop of at least 20 g L−1or requiring transfusion of 2 U or more of either whole blood or packed red blood cells | Up to 90days |
| Arabi 2020 (2) | - VTE detection during ICU stay including upper limb and neck ultrasonography, spiral computed tomography (CT) to evaluate for PE, ventilation–perfusion (V/Q) scan of the lungs, CT scan of the abdomen to evaluate thrombosis | 90-day |
| Allen 2016 (3) | - Pulmonary embolisms were diagnosed with CT angiography after symptoms of hypoxemia, tachycardia, or both. Pulmonary embolism was defined as a filling defect of pulmonary vasculature on CT angiography - Patients were considered positive for DVT if abnormalities were detected in the proximal venous system (above or including the popliteal vein). Calf veins were not included | ~4 years |
| Haut 2007 (4) | - PE identified by either ventilation-perfusion scan or computed tomography (CT) angiogram | NR |
| Shackford 2016 (5) | - Used CLOTT Study Group definition: diagnosis of DVT was made if there was a dilated noncompressible vein, an echogenic thrombus, or absence of color flow characteristics, which included a lack of spontaneity, phasicity, pulsatility, and augmentability as described in the American Thoracic Society guidelines - diagnosis of PE was made in symptomatic patients by computed tomography pulmonary angiogram (CT/PE) protocol, | 1 year |

**REFERENCES:**

1. Kay AB, Morris DS, Woller SC, Stevens SM, Bledsoe JR, Lloyd JF, et al. Trauma patients at risk for venous thromboembolism who undergo routine duplex ultrasound screening experience fewer pulmonary emboli: A prospective randomized trial. Journal of Trauma and Acute Care Surgery. 2021;90(5):787-96.

2. Arabi YM, Burns KE, Alsolamy SJ, Alshahrani MS, Al-Hameed FM, Arshad Z, et al. Surveillance or no surveillance ultrasonography for deep vein thrombosis and outcomes of critically ill patients: a pre-planned sub-study of the PREVENT trial. Intensive care medicine. 2020;46(4):737-46.

3. Allen CJ, Murray CR, Meizoso JP, Ginzburg E, Schulman CI, Lineen EB, et al. Surveillance and early management of deep vein thrombosis decreases rate of pulmonary embolism in high-risk trauma patients. Journal of the American College of Surgeons. 2016;222(1):65-72.

4. Haut ER, Noll K, Efron DT, Berenholz SM, Haider A, Cornwell III EE, et al. Can increased incidence of deep vein thrombosis (DVT) be used as a marker of quality of care in the absence of standardized screening? The potential effect of surveillance bias on reported DVT rates after trauma. Journal of Trauma and Acute Care Surgery. 2007;63(5):1132-7.

5. Shackford SR, Cipolle MD, Badiee J, Mosby DL, Knudson MM, Lewis PR, et al. Determining the magnitude of surveillance bias in the assessment of lower extremity deep venous thrombosis: a prospective observational study of two centers. Journal of Trauma and Acute Care Surgery. 2016;80(5):734-41.
