# Supplementary material for: Ultrasound surveillance for deep venous thrombosis and subsequent venous thromboembolism in adults with trauma: A systematic review and meta-analysis
Source: Medicine (Baltimore). 2023 Oct 27;102(43):e35625. doi: 10.1097/MD.0000000000035625 (PMC10615543; doi:10.1097/MD.0000000000035625)
Supplement: Supplementary file 5 [file medi-102-e35625-s005.docx]

**Supplemental Digital Content File 4:**

**Search Strategy**

Databases: Ovid Medline [ppezv], Embase [oemezd]; Cochrane Library Controlled Clinical Trials Registry (CENTRAL)

Grey Literature: To be searched by guideline development team

Filters: RCTs and Systematic Reviews (modified CADTH), Studies/reports of humans

Preferred Reference Output: XML

Search Alerts Required: TBD

Search Methods:

“Electronic Search Strategy

The literature search was performed by an information specialist following PRISMA-S guidance (reference: Rethlefsen ML, et al. PRISMA-S: an extension to the PRISMA Statement for Reporting Literature Searches in Systematic Reviews. Syst Rev. 2021 Jan 26;10(1):39. https://doi.org/10.1186/s13643-020-01542-z.) and using a peer-reviewed search strategy (Appendix 1). The search strategy was reviewed according to the methods described in McGowan, 2016 (reference: Jessie McGowan, et al. PRESS Peer Review of Electronic Search Strategies: 2015 Guideline Statement. J Clin Epi. 2016(75):40-46. https://doi.org/10.1016/j.jclinepi.2016.01.021.).

Published literature was identified by searching the following bibliographic databases on October 19, 2021: MEDLINE (1946–) with in-process records and daily updates via Ovid; Embase (1974–) via Ovid; The Cochrane Library via Wiley. The search strategy consisted of both controlled vocabulary, such as the National Library of Medicine’s MeSH (Medical Subject Headings), and keywords.

Concept #2: Venous Thromboembolism

9 exp Venous Thrombosis/ use ppez 57099

10 exp Vein Thrombosis/ use oemezd 139295

11 exp Pulmonary Embolism/ use ppez 41002

12 exp Lung Embolism/ use oemezd 102069

13 ((vein* or ven*) and (thrombo* or embol*)).ti,ab,kw. 355855

14 ((blood or lung or lungs or pulmonary) adj3 (clot* or thrombo* or embol*)).ti,ab,kw. 169668

15 (VTE or DVT or PE or VTEs or DVTs or PEs).ti,ab,kw. 194862

16 (pulmonary and (embolism or embolisms or thromboembolism or thrombo-embolism or thromboembolisms or thrombo-embolisms)).ti,ab,kw. 112579

17 (thrombus* or thrombotic* or thrombolic* or thromboemboli* or thrombos* or embol*).ti,ab,kw. 896109

18 exp Embolism/ 216375

19 exp Thromboembolism/ 580485

20 exp Thrombosis/ 480059

21 Thrombophlebitis/ 33552

22 or/9-21 1313682

Concept #3: VTE prophylaxis/prevention

32 Thrombosis/pc 21073

33 venous thromboembolism/pc use ppez 5008

34 exp vein thrombosis/pc use oemezd 11886

35 pulmonary embolism/pc use ppez 5070

36 exp lung embolism/pc use oemezd 5961

37 post-exposure prophylaxis/ 4981

38 (chemoprevention or chemo-prevention or chemoprophylaxis or chemo-prophylaxis or thromboprophylaxis or thrombo-prophylaxis).ti,ab,kw. 57149

39 or/32-38 102063

Concept #6: Trauma

9 exp "Wounds and Injuries"/ use ppez 950785

10 exp Injury/ use oemezd 2341382

11 ((abdominal or abdomen or thorax or thoracic) adj3 (injur* or trauma* or perforat* or penetrat*)).ti,ab,kw. 48470

12 ((splenic or spleen) adj3 rupture*).ti,ab,kw. 7869

13 ((stomach or gastric) adj3 (rupture or perforation or injur* or burst*)).ti,ab,kw. 13760

14 ((stab* or gunshot or shot or penetrat* wound* or bullet?) adj3 (abdomen* or abdominal or stomach or splenic or spleen or thorax or thoracic)).ti,ab,kw. 4747

15 (asphyxia or burn* or drown or drowning or fracture* or frostbite or injur* or lacerat* or perforation or rupture or stab* or shot or shoot* or trauma* or wound*).ti,ab,kw. 6576386

16 or/9-15 7897678

Concept #10: Surveillance

31 exp Mass Screening/ 404226

32 mandatory testing/ 1765

33 multiphasic screening/ 1127

34 exp early diagnosis/ 174258

35 Diagnostic tests, Routine/ 95904

36 Risk Assessment/ 925427

37 (screen or screens or screening or surveil or surveillance or tested or testing).ti,ab,kw. 5397975

38 ((early or rapid* or routin*) adj6 (detect* or diagnos* or identif* or test*)).ti,ab,kf. 1118480

39 (drive adj6 (diagnos* or detect* or test* or identif*)).ti,ab,kf. 7305

40 ((point of care or poc) adj6 (detect* or diagnos* or identif* or test*)).ti,ab,kf. 34221

41 (risk? adj1 assess*).ti,ab,kf. 216483

42 or/31-41 7245494

Concept #11: Duplex doppler ultrasonography

44 exp Ultrasonography, Doppler, Duplex/ 30261

45 (doppler adj duplex adj ultrasonography).ti,ab,kw. 136

46 blood flow velocity.ti,ab,kw. 19115

47 or/44-46 48892

√
