# Supplementary material for: Ultrasound surveillance for deep venous thrombosis and subsequent venous thromboembolism in adults with trauma: A systematic review and meta-analysis
Source: Medicine (Baltimore). 2023 Oct 27;102(43):e35625. doi: 10.1097/MD.0000000000035625 (PMC10615543; doi:10.1097/MD.0000000000035625)
Supplement: Supplementary file 6 [file medi-102-e35625-s006.docx]

**Supplemental Digital Content Table 2: Predefined data abstraction sheet**

| Basic Study Information | |
| --- | --- |
| Study Title |  |
| Author |  |
| Year of Publication |  |
| Correspondence Email |  |
| Number of Sites |  |
| Country/Countries of Study |  |
| Population Description | |
| Inclusion Criteria |  |
| Exclusion Criteria |  |
| Median/Mean age |  |
| Number of Patients Fulfilling Criteria and approached? (for RCT) |  |
| Number of patients randomized? (for RCT) |  |
| Intervention and control | |
| Type of screening US |  |
| frequency of screening US |  |
| Other cointerventions | |
| Pharmacological prophylaxis regimen (dose and time of initiation) |  |
| Mechanical prophylaxis |  |
| Risk of Bias Assessment (RCT) | |
| Randomization and sequence generation? |  |
| Concealment of the intervention? |  |
| Blinding of the intervention? |  |
| Were there any missing data? |  |
| Was there a concern of selective reporting? |  |
| Any other bias noted?  Risk of bias overall? |  |
| Risk of Bias Assessment (observational studies) | |
| Bias due to confounding |  |
| selection of participants into the study, |  |
| classification of interventions |  |
| deviations from intended interventions |  |
| missing data, |  |
| measurement of outcomes |  |
| selection of the reported result |  |
| Outcome #1 (Repeated for each outcome) | |
| Outcome being evaluated |  |
| Dichotomous or continuous outcome |  |
| Intervention 1: Number analyzed |  |
| Intervention 1: Number of Events/Mean |  |
| Intervention 2: Number analyzed |  |
| Intervention 2: Number of Events/Mean |  |
|  |  |
