# Supplementary material for: Ultrasound surveillance for deep venous thrombosis and subsequent venous thromboembolism in adults with trauma: A systematic review and meta-analysis
Source: Medicine (Baltimore). 2023 Oct 27;102(43):e35625. doi: 10.1097/MD.0000000000035625 (PMC10615543; doi:10.1097/MD.0000000000035625)
Supplement: Supplementary file 7 [file medi-102-e35625-s007.docx]

**Supplemental Digital Content Table 3: Details of the eligible trials**

| Study | Study aim | N, Population, inclusion and exclusion | Type of screening US and frequency | PVTEPx | Mechanical VTEPx | **Mortality** | | | | DVT proximal and distal, time to DVT Dx | | | | PE and time to PE Dx | | | |  |
| --- | --- | --- | --- | --- | --- | --- | --- | --- | --- | --- | --- | --- | --- | --- | --- | --- | --- | --- |
|  |  |  |  |  |  | **US screen** | | **No US screen** | | US screen | | No US screen | | US screen | | No US screen | |  |
|  |  |  |  |  |  | **Event** | **Total** | **Event** | **Total** | Event | Total | Event | Total | Event | Total | Event | Total |  |
| **RCT** | | | | | | | | | | | | | | | | | |  |
| Kay 2021 (1) | Assess the value of a routine DUS protocol among at-risk trauma patients | N= 1,989  Adults with trauma and moderate- or high-risk defined by a RAP score of ≥5  97% has blunt trauma  Excluded Pts with hypercoagulable state and APS, or Hx of VTE within 6 ms | **US group** Serial whole-leg DUS scans on hospital days 1, 3, and 7 and weekly thereafter until hospital discharge.  No US for upper extremities and neck  **No US**  US at discretion of trauma provider based on clinical suspicion of VTE | Was equal between group  75% on LWMH  20% UFH  2-3% DOACs | NR | **In-hospital mortality** | | | | **Proximal DVT , In-hospital** | | | | **PE , In-hospital** | | | |  |
|  |  |  |  |  |  | 25 | 995 | 34 | 994 | 19 | 995 | 8 | 994 | 1 | 995 | 9 | 994 |  |
|  |  |  |  |  |  | **90 day mortality** | | | | **Proximal DVT , 90 day** | | | | **PE , 90 day** | | | |  |
|  |  |  |  |  |  | 56 | 995 | 67 | 994 | 1 | 995 | 1 | 994 | 5 | 995 | 6 | 994 |  |
|  |  |  |  |  |  |  |  |  |  | **Distal DVT , In-hospital** | | | | **Time to PE Dx, median IQR** | | | |  |
|  |  |  |  |  |  |  |  |  |  | 124 | 995 | 8 | 994 | 15 | 1 | 7.1 (5.2) | 9 |  |
|  |  |  |  |  |  |  |  |  |  | **Distal DVT , 90 day** | | | |  |  |  |  |  |
|  |  |  |  |  |  |  |  |  |  | 3 | 995 | 7 | 994 |  |  |  |  |  |
|  |  |  |  |  |  |  |  |  |  | **Distal DVT propagation, In-hospital** | | | |  |  |  |  |  |
|  |  |  |  |  |  |  |  |  |  | 3 | 995 | 1 | 994 |  |  |  |  |  |
|  |  |  |  |  |  |  |  |  |  | **Distal DVT propagation, 90 day** | | | |  |  |  |  |  |
|  |  |  |  |  |  |  |  |  |  | 0 | 995 | 0 | 994 |  |  |  |  |  |
|  |  |  |  |  |  |  |  |  |  | **Time to Distal DVT Dx, median IQR** | | | |  |  |  |  |  |
|  |  |  |  |  |  |  |  |  |  | 1.41 (2.5) | 124 | 2.96 (3.8) | 8 |  |  |  |  |  |
|  |  |  |  |  |  |  |  |  |  | **Time to proximal DVT Dx, median IQR** | | | |  |  |  |  |  |
|  |  |  |  |  |  |  |  |  |  | 0.69 (1.8) | 19 | 2.94 (4.9) | 8 |  |  |  |  |  |
| **Observational studies** | | | | | | | | | | | | | | | | | |  |
| Allen 2016 (2) | analyze institution’s experience with routine surveillance at high risk for VTE in an effort to reduce the rate of PE | N= 402 asymptomatic trauma patients were identified as high risk for VTE RAP>10  70-80 % blunt , 40 % TBI in both groups  majority of US group required surgical intervention, w sustained vascular injury and SCI | **US group**  Bilateral VDU weekly of both LE were examined from the ankle to the inguinal ligaments proximal venous system (above or including the popliteal vein). Calf veins, Neck and upper extremity were not included.  **non-surve US**  If Sx indicated clinical suspicion of DVT ( leg  swelling or pain | About 9-10 % didn’t receive any  90% received  UFH ~40-50 %  and  Enox 7-16 % and was started around 3^rd^ hospital day | SCD were used if not prohibited by plaster immobilizer or external fixators | 35 | 259 | 22 | 143 | 30 | 259 | 3 | 143 | 5 | 259 | 10 | 143 |  |
|  |  |  |  |  |  |  |  |  |  |  |  |  |  |  |  |  |  |  |
| Haut 2007 (3) | examine the influence of duplex scanning on the incidence of DVT | 7559  Before and after study  US group are less injured and less likely to have penetrating injuries | **US group**  Pts admitted (1999–2005) after DUS screening For asymptomatic trauma pts at “high risk” for DVT ^#^  **non- US**  pts admitted (1995–1997) before DUS screening | enoxa (dosed 30 mg twice daily) was used but NR per group | NR | 0 | 5561 | 1 | 1389 | 39 | 5561 | 1 | 1389 | 18 | 5561 | 1 | 1389 |  |
| Shack-ford 2016 (4) | Determine magnitude of surveillance bias associated with LE DUS | N= 1226 Comparison between 2 hospitals w different US screening strategies  overall Injury Severity Score (ISS), VTE risk between the two groups was similar.  Included neurotrauma pts TBI and SCI | **US group “serial LE DUS @ Scripps Mercy hospital”**  twice weekly for patients admitted to ICU, weekly for patients admitted to the trauma floor  **non-surve US**  **“@ Christiana Care”**  Used LE DUS only for symptomatic pts | significantly different between 2 groups    57% in US group Scripps Mercy hospital  80% in non-surve US  Christiana Care | significantly different between 2 groups  94% used IPC in US group Scripps Mercy hospital  60% used IPC in non-surve US  Christiana Care | NR | NR | NR | NR | **Proximal “Above-knee DVT”** | | | | 3 | 772 | 2 | 454 |  |
|  |  |  |  |  |  |  |  |  |  | 12 | 772 | 2 | 454 |  |  |  |  |  |
|  |  |  |  |  |  |  |  |  |  | **Distal** **“below-knee DVT”** | | | |  |  |  |  |  |
|  |  |  |  |  |  |  |  |  |  | 55 | 772 | 6 | 454 |  |  |  |  |  |
| Arabi 2020 **Sub‑**  **study of PREVENT trial (5)** | examine association between surveillance for DVT by twice weekly US and 90-day all-cause mortality | N= 2,065 Trauma pts 8.5 % surveillance group and 7.1 % non‑surveillance group🡪  **will be downgraded for indirectness by 1 points as population is not only trauma.** | **US group**  All proximal, distal, LL, UL, and neck US within 48 h then Twice weekly as part of PREVENT RCT  **non-surve US**  requested by the treating team based on clinical suspicion. | Was equal between group  ~60-65% UFH  ~ 35-40% LMWH | IPC use in surveillance group was 54.6%  IPC use in non-surveillance group was 30% | **90- day mortality** | | | | **All DVT (proximal and distal)** | | | | **PE** | | | |  |
|  |  |  |  |  |  | 424 | 1682 | 83 | 369 | 162 | 1682 | 10 | 382 | 16 | 1682 | 6 | 382 |  |
|  |  |  |  |  |  | **ICU mortality** | | | | **Time to DVT Dx, median IQR** | | | | **Time to PE Dx, median IQR** | | | |  |
|  |  |  |  |  |  | 245 | 1682 | 61 | 383 | 4 d (2-10) | 1682 | 20 d  (16-22) | 382 | 4 d (2.5-5) | 1682 | 7.5 d (6.1-28.9) | 382 |  |
|  |  |  |  |  |  | **Hospital mortality** | | | |  |  |  |  |  |  |  |  |  |
|  |  |  |  |  |  | 439 | 1682 | 83 | 383 |  |  |  |  |  |  |  |  |  |

**Abbreviation**: DUS= duplex ultrasound surveillance, RAP= risk assessment profile =, RR= retrospective review , PVTEPx= Pharmacological VTE prophylaxis , NR= not reported, VDU= venous duplex ultrasound

^#^ based upon the following risk factors: age>40 years, pelvic or femur or tibial fracture, venous injury, femoral venous catheter placement, spinal cord injury, severe traumatic brain injury (Glasgow Coma Score<8), or anticipated need for prolonged immobilization (>3 days).

**Sub‑ study of PREVENT trial Cox proportional hazards model added separately in GRADE evidence profile**

- **90 day mortality** : Generalized linear mixed model aOR (95% CI): 0.87 (0.63, 1.20), Cox proportional hazards model aHR (95% CI): 0.75 (0.57, 0.99)**,** Cox proportional hazards model with bootstrapping, aHR (95% CI): 0.75 (0.57, 0.98)
- **ICU mortality**: Generalized linear mixed model aOR (95% CI): 0.64 (0.44, 0.92**),** Cox proportional hazards model aHR (95% CI): 0.71 (0.51, 0.99), Cox proportional hazards model with bootstrapping, aHR (95% CI): 0.69 (0.51, 0.97)
- **Hospital mortality:** Generalized linear mixed model aOR (95% CI): 0.95 (0.69, 1.32), Cox proportional hazards model aHR (95% CI): 0.78 (0.59, 1.02), Cox proportional hazards model with bootstrapping, aHR (95% CI): 0.77 (0.58, 1.04)
- **DVT:** Generalized linear mixed model aOR (95% CI): 3.64 (1.82, 7.28), Cox proportional hazards model aHR (95% CI): 5.22 (2.56, 10.63), Cox proportional hazards model with bootstrapping, aHR (95% CI): 5.49 (2.92, 13.02)
- **PE:** Generalized linear mixed model aOR (95% CI): 0.28 (0.08, 1.00), Cox proportional hazards model aHR (95% CI): 0.53 (0.20, 1.36), Cox proportional hazards model with bootstrapping, aHR (95% CI): 0.56 (0.19, 1.91)

**References:**

1. Kay AB, Morris DS, Woller SC, Stevens SM, Bledsoe JR, Lloyd JF, et al. Trauma patients at risk for venous thromboembolism who undergo routine duplex ultrasound screening experience fewer pulmonary emboli: A prospective randomized trial. Journal of Trauma and Acute Care Surgery. 2021;90(5):787-96.

2. Allen CJ, Murray CR, Meizoso JP, Ginzburg E, Schulman CI, Lineen EB, et al. Surveillance and early management of deep vein thrombosis decreases rate of pulmonary embolism in high-risk trauma patients. Journal of the American College of Surgeons. 2016;222(1):65-72.

3. Haut ER, Noll K, Efron DT, Berenholz SM, Haider A, Cornwell III EE, et al. Can increased incidence of deep vein thrombosis (DVT) be used as a marker of quality of care in the absence of standardized screening? The potential effect of surveillance bias on reported DVT rates after trauma. Journal of Trauma and Acute Care Surgery. 2007;63(5):1132-7.

4. Shackford SR, Cipolle MD, Badiee J, Mosby DL, Knudson MM, Lewis PR, et al. Determining the magnitude of surveillance bias in the assessment of lower extremity deep venous thrombosis: a prospective observational study of two centers. Journal of Trauma and Acute Care Surgery. 2016;80(5):734-41.

5. Arabi YM, Burns KE, Alsolamy SJ, Alshahrani MS, Al-Hameed FM, Arshad Z, et al. Surveillance or no surveillance ultrasonography for deep vein thrombosis and outcomes of critically ill patients: a pre-planned sub-study of the PREVENT trial. Intensive care medicine. 2020;46(4):737-46.
