# Supplementary figures and images for: Ultrasound surveillance for deep venous thrombosis and subsequent venous thromboembolism in adults with trauma: A systematic review and meta-analysis
Source: Medicine (Baltimore). 2023 Oct 27;102(43):e35625. doi: 10.1097/MD.0000000000035625 (PMC10615543; doi:10.1097/MD.0000000000035625)

**Supplemental Digital Content Figure 1: Risk of bias assessment of the eligible studies**


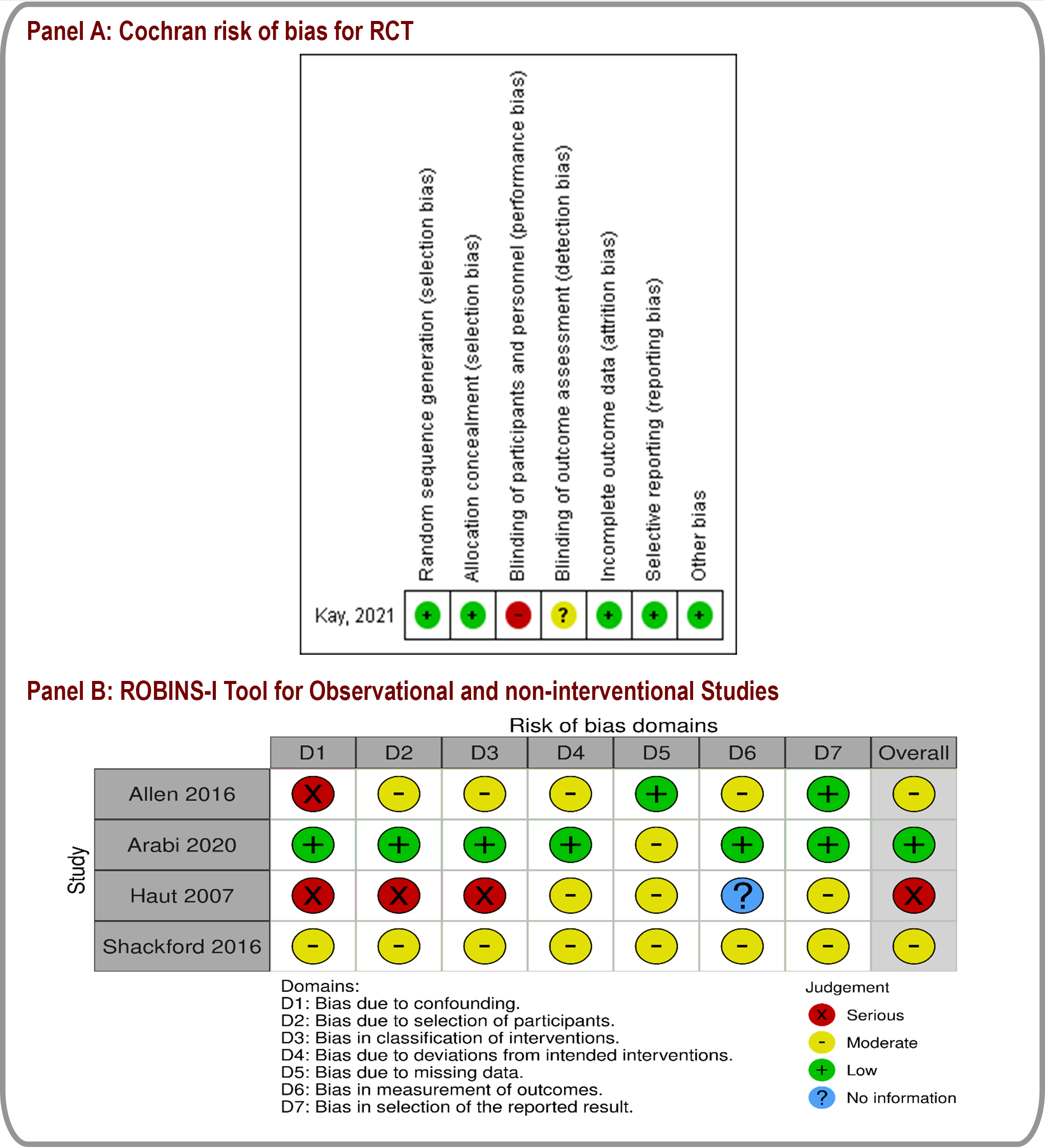

Supplement: Supplementary file 8 [file medi-102-e35625-s008.docx]

**Supplemental Digital Content Figure 3: Sensitivity analysis for low-moderate ROB studies.**


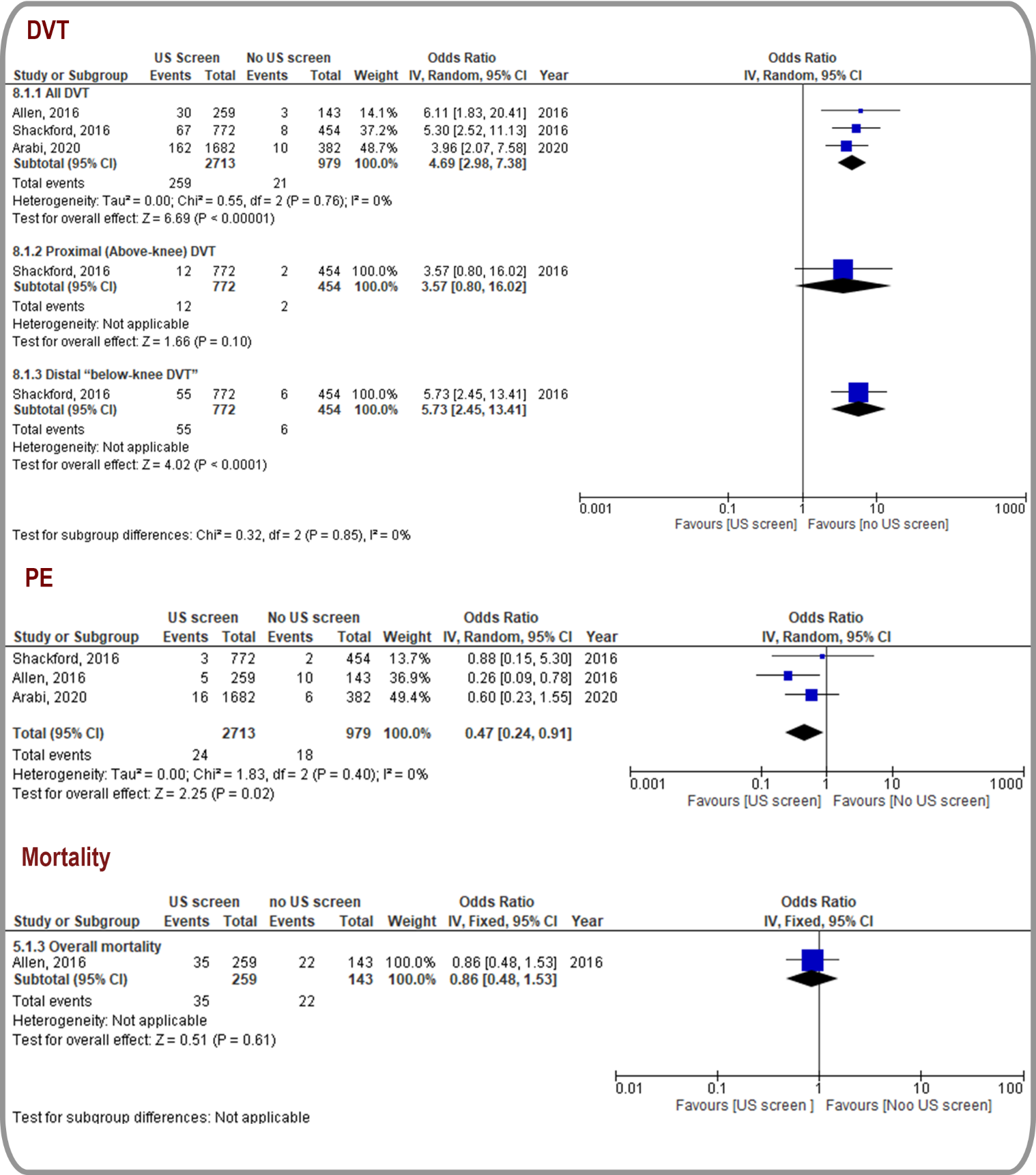

Supplement: Supplementary file 11 [file medi-102-e35625-s011.docx]

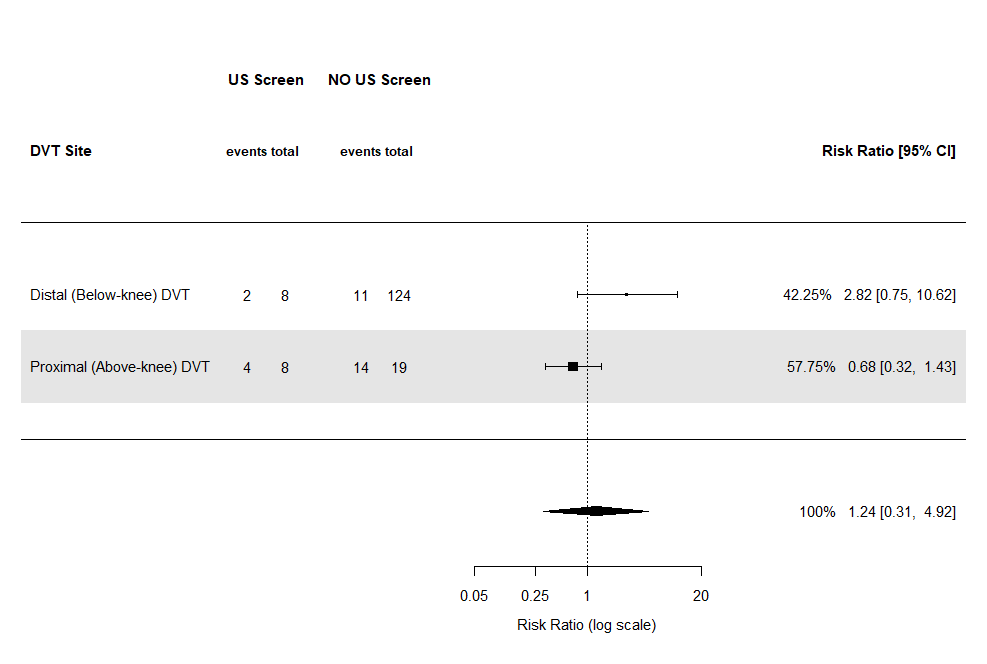

Supplement: Supplementary file 12 [file medi-102-e35625-s012.tif]
