# Supplementary material for: Ultrasound surveillance for deep venous thrombosis and subsequent venous thromboembolism in adults with trauma: A systematic review and meta-analysis
Source: Medicine (Baltimore). 2023 Oct 27;102(43):e35625. doi: 10.1097/MD.0000000000035625 (PMC10615543; doi:10.1097/MD.0000000000035625)
Supplement: Supplementary file 9 [file medi-102-e35625-s009.docx]

**Supplemental Digital Content Table 4: Evidence profile including GRADE certainty assessments**

| **Certainty assessment** | | | | | | | **№ of patients** | | **Effect** | | **Certainty** | **Importance** |
| --- | --- | --- | --- | --- | --- | --- | --- | --- | --- | --- | --- | --- |
| **№ of studies** | **Study design** | **Risk of bias** | **Inconsistency** | **Indirectness** | **Imprecision** | **Other considerations** | **we recommend routine VTE ultrasonography (US) screening** | **No routine surveillance** | **Relative (95% CI)** | **Absolute (95% CI)** |  |  |
| **90-day mortality-RCT (follow-up: 90 days)** | | | | | | | | | | | | |
| 1 | randomised trials | not serious^a^ | not serious^b^ | serious^c^ | serious^d^ | none^e^ | 56/995 (5.6%) | 67/994 (6.7%) | **RR 0.83** (0.59 to 1.18) | **11 fewer per 1,000** (from 28 fewer to 12 more) | ⨁⨁◯◯ Low | CRITICAL |
| **In-hospital mortality-RCT** | | | | | | | | | | | | |
| 1 | randomised trials | not serious^a^ | not serious^b^ | serious^c^ | very serious^f^ | none^e^ | 25/995 (2.5%) | 34/994 (3.4%) | **RR 0.73** (0.44 to 1.22) | **9 fewer per 1,000** (from 19 fewer to 8 more) | ⨁◯◯◯ Very low | CRITICAL |
| **90-day mortality ( PREVENT sub-study) (assessed with: Cox proportional hazards model )** | | | | | | | | | | | | |
| 1 | observational studies | not serious | not serious^b^ | serious^c,g^ | serious^h^ | none | 424/1682 (25.2%) | 83/369 (22.5%) | **HR 0.75** (0.57 to 0.99) | **51 fewer per 1,000** (from 90 fewer to 2 fewer) | ⨁◯◯◯ Very low | CRITICAL |
|  |  |  |  |  |  |  |  | 11.0%^i^ |  | **26 fewer per 1,000** (from 46 fewer to 1 fewer) |  |  |
|  |  |  |  |  |  |  |  | 14.0%^i^ |  | **33 fewer per 1,000** (from 58 fewer to 1 fewer) |  |  |
| **ICU mortality ( PREVENT sub-study) (assessed with: Cox proportional hazards model )** | | | | | | | | | | | | |
| 1 | observational studies | not serious | not serious^b^ | serious^c,g^ | serious^h^ | none | 245/1682 (14.6%) | 61/383 (15.9%) | **HR 0.71** (0.51 to 0.99) | **43 fewer per 1,000** (from 75 fewer to 1 fewer) | ⨁◯◯◯ Very low | CRITICAL |
|  |  |  |  |  |  |  |  | 11.0%^i^ |  | **31 fewer per 1,000** (from 52 fewer to 1 fewer) |  |  |
|  |  |  |  |  |  |  |  | 14.0%^i^ |  | **38 fewer per 1,000** (from 66 fewer to 1 fewer) |  |  |
| **In hospital mortality PREVENT sub-study (assessed with: Cox proportional hazards model)** | | | | | | | | | | | | |
| 1 | observational studies | not serious | not serious^b^ | serious^c,g^ | serious^d^ | none | 439/1682 (26.1%) | 83/383 (21.7%) | **HR 0.78** (0.59 to 1.02) | **43 fewer per 1,000** (from 83 fewer to 4 more) | ⨁◯◯◯ Very low | CRITICAL |
|  |  |  |  |  |  |  |  | 11.0%^i^ |  | **23 fewer per 1,000** (from 44 fewer to 2 more) |  |  |
|  |  |  |  |  |  |  |  | 14.0%^i^ |  | **29 fewer per 1,000** (from 55 fewer to 3 more) |  |  |
| **In-Hospital distal DVT- RCT** | | | | | | | | | | | | |
| 1 | randomised trials | not serious^j^ | not serious^b^ | serious^c^ | serious^k^ | none^e^ | 124/995 (12.5%) | 8/994 (0.8%) | **RR 15.48** (7.62 to 31.48) | **117 more per 1,000** (from 53 more to 245 more) | ⨁⨁◯◯ Low | IMPORTANT |
| **In-Hospital proximal DVT-RCT** | | | | | | | | | | | | |
| 1 | randomised trials | not serious^j^ | not serious^b^ | serious^c^ | very serious^f^ | none^e^ | 19/995 (1.9%) | 8/994 (0.8%) | **RR 2.37** (1.04 to 5.39) | **11 more per 1,000** (from 0 fewer to 35 more) | ⨁◯◯◯ Very low | CRITICAL |
| **In-hospital, Distal DVT propagation-RCT** | | | | | | | | | | | | |
| 1 | randomised trials | not serious^j^ | not serious^b^ | serious^c^ | very serious^f^ | none^e^ | 3/995 (0.3%) | 1/994 (0.1%) | **RR 3.00** (0.31 to 28.76) | **2 more per 1,000** (from 1 fewer to 28 more) | ⨁◯◯◯ Very low | CRITICAL |
| **All DVT- observational** | | | | | | | | | | | | |
| 4 | observational studies | serious^l^ | not serious^m^ | serious^c^ | not serious | none | 298/8274 (3.6%) | 22/2368 (0.9%) | **OR 4.87** (3.13 to 7.57) | **34 more per 1,000** (from 19 more to 57 more) | ⨁◯◯◯ Very low | CRITICAL |
| **All DVT - observation using pooled adjusted OR** | | | | | | | | | | | | |
| 2 | observational studies | not serious | not serious | serious^c,g^ | not serious | none | 0/0 | 1.0%^6,n^ | **OR 4.49** (2.76 to 7.31) | **33 more per 1,000** (from 17 more to 59 more) | ⨁◯◯◯ Very low | CRITICAL |
|  |  |  |  |  |  |  |  | 7.0%^n^ |  | **183 more per 1,000** (from 102 more to 285 more) |  |  |
| **All DVT- PREVENT sub study (assessed with: Cox proportional hazards model )** | | | | | | | | | | | | |
| 1 | observational studies | not serious | not serious^b^ | serious^c,g^ | serious^k^ | none | 162/1682 (9.6%) | 10/382 (2.6%) | **HR 5.22** (2.56 to 10.63) | **103 more per 1,000** (from 39 more to 220 more) | ⨁◯◯◯ Very low | CRITICAL |
|  |  |  |  |  |  |  |  | 12.0%^o^ |  | **367 more per 1,000** (from 159 more to 623 more) |  |  |
| **In-hospital PE -RCT** | | | | | | | | | | | | |
| 1 | randomised trials | not serious^j^ | not serious^b^ | serious^c^ | very serious^f^ | none^e^ | 1/995 (0.1%) | 9/994 (0.9%) | **RR 0.11** (0.01 to 0.87) | **8 fewer per 1,000** (from 9 fewer to 1 fewer) | ⨁◯◯◯ Very low | CRITICAL |
| **90 d PE- RCT (follow-up: 90 days)** | | | | | | | | | | | | |
| 1 | randomised trials | not serious^j^ | not serious^b^ | serious^c^ | very serious^f^ | none^e^ | 5/995 (0.5%) | 6/994 (0.6%) | **RR 0.83** (0.25 to 2.72) | **1 fewer per 1,000** (from 5 fewer to 10 more) | ⨁◯◯◯ Very low | CRITICAL |
| **PE observation using pooled adjusted OR** | | | | | | | | | | | | |
| 2 | observational studies | not serious | not serious | serious^c,g^ | serious^d^ | none | 0/0 | 3.0%^p^ | **OR 0.65** (0.28 to 1.52) | **10 fewer per 1,000** (from 21 fewer to 15 more) | ⨁◯◯◯ Very low | CRITICAL |
| **PE observational studies (low-moderate ROB)** | | | | | | | | | | | | |
| 3 | observational studies | serious^q^ | not serious^r^ | serious^c^ | serious^s^ | none | 24/2713 (0.9%) | 18/979 (1.8%) | **OR 0.47** (0.24 to 0.91) | **10 fewer per 1,000** (from 14 fewer to 2 fewer) | ⨁◯◯◯ Very low | CRITICAL |
| **PE- PREVENT sub study (assessed with: Cox proportional hazards model )** | | | | | | | | | | | | |
| 1 | observational studies | not serious | not serious^b^ | very serious^c,g^ | very serious^f^ | none | 16/1682 (1.0%) | 6/382 (1.6%) | **HR 0.53** (0.20 to 1.40) | **7 fewer per 1,000** (from 13 fewer to 6 more) | ⨁◯◯◯ Very low | CRITICAL |
|  |  |  |  |  |  |  |  | 11.0%^o^ |  | **50 fewer per 1,000** (from 87 fewer to 41 more) |  |  |
| **Time to distal DVT Dx -RCT (assessed with: days)** | | | | | | | | | | | | |
| 1 | randomised trials | not serious^j^ | not serious^b^ | serious^c^ | very serious^t^ | none | 124 | 8 | - | MD **1.55 lower** (4.22 lower to 1.12 higher) | ⨁◯◯◯ Very low | IMPORTANT |
| **Time to proximal DVT Dx -RCT (assessed with: days)** | | | | | | | | | | | | |
| 1 | randomised trials | not serious^j^ | not serious^b^ | serious^c^ | very serious^t^ | none | 19 | 8 | - | MD **2.25 lower** (5.74 lower to 1.24 higher) | ⨁◯◯◯ Very low | IMPORTANT |
| **Complications-RCT** | | | | | | | | | | | | |
| 1 | randomised trials | serious^u^ | not serious^b^ | serious^c^ | serious^s^ | none | Distal DVT: 2 (25.0%) of 8 subjects in the NoUs group and 11 (8.9%) of 124 subjects in the US group received full-dose anticoagulation (p = 0.18).  Proximal DVT: full Anticoagulation was initiated in 4 (50.0%) of 8 subjects in the NoUS group compared with 14 (73.7%) of 19 subjects in the US group (p = 0.38).  No difference in major bleeding rate between US and noUS group | | | | ⨁◯◯◯ Very low | IMPORTANT |

**CI:** confidence interval; **HR:** hazard Ratio; **MD:** mean difference; **OR:** odds ratio; **RR:** risk ratio

#### Explanations

a. All studies lack blinding of participants and personnel. However, ROB was not graded down as outcome (mortality) was objective and blinding is irrelevant for mortality

b. Only 1 study reporting data - unable to compare consistency across studies

c. Downgraded by one level as the PICO question in the included study is slightly different from our PICO. Our PICO focused on trauma patients who are not candidate for pharma VTEPx vs included study ( pts were on pharma VTEPx but at high risk or VTE)

d. Downgraded by 1 level as CI is wide does not exclude potentially insignificant differences

e. Only 1 study reporting data - unable to assess publication bias across studies

f. Downgraded by 2 level as the total numbers of events was small and confidence interval is wide to reliably estimate an effect

g. PREVENT sub-study was performed in a wide variety of patients (medical and surgical). Trauma accounted for less than the majority of patients included (about 8% in both US and no US surveillance group)

h. Downgraded by 1 level as the upper limit of CI approaching no effect

i. Baseline mortality rate based on observational data was 11-14%. Al-Hameed FM, Al-Dorzi HM, Qadhi AI, Shaker A, Al-Gahtani FH, Al-Jassir FF, Zahir GF, Al-Khuwaitir TS, Addar MH, Al-Hajjaj MS, Abdelaal MA, Aboelnazar EY. Thromboprophylaxis and mortality among patients who developed venous thromboembolism in seven major hospitals in Saudi Arabia. Ann Thorac Med. 2017

j. RCT was unblinded (participants, personnel, and unclear blinding of outcome assessors). However, ROB was not downgraded a since all RCT used protocolized method for VTE diagnosis and less likely to be affected by lack of blinding

k. Downgraded by 1 level for wide CI

l. We downgraded the quality of evidence by one level for serious baseline differences between the groups, not adjusted for in analysis with likely residual confounding. Sensitivity analysis for low-moderate ROB studies ( excluding Haut 2007) showed similar results

m. Didn’t down grade as all point estimates and confidence intervals favor no US group. Excluding high ROB study ( Haut 2007) gave similar point estimate (OR 4.69, 95% CI 2.98 to 7.38)

n. This is the baseline risk in the absence of prophylaxis. The risk of DVT and PE in patients with trauma can be considerable and has been reported in 5%- 63% of patients, respectively depending on patient’s risk factors, modality of prophylaxis, and methods of detection . DVT may be higher (17%) among patients with TBI and even higher in SCI

o. the overall incidence of DVT and PE was reported in review (n=3005 patients) to be around 12% and 11%, respectively (Geerts et al., 1994).

p. Baseline VTE risk in isolated TBI patients reported by Denson et al [Denson K, et al. Incidence of venous thromboembolism in patients with traumatic brain injury. Am J Surg. 2007;193:380–3]

q. We downgraded the quality of evidence by one level for serious baseline differences between the groups, not adjusted for in analysis with likely residual confounding. majority of US group required surgical intervention, w sustained vascular injury and SCI

r. Didn’t down grade as all point estimates and confidence intervals favor US group. Including high ROB study ( Haut 2007) gave point estimate (OR 0.69, 95% CI 0.26 to 1.85) with I2 52% and removing this study in sensitivity analysis removed heterogeneity.

s. downgraded by 1 level due to too few events to reliably estimate an effect.

t. We downgraded by 2 level for serious imprecision due to wide confidence interval. Time to proximal and distal DVT Dx reported as median IQR in original publication while in fact it represents mean SD

u. We downgraded by 1 level for serious risk of bias. RCT was unblinded (participants, personnel, and unclear blinding of outcome assessors), and this outcome is somewhat subjective
