# Supplementary material for: Ultrasound surveillance for deep venous thrombosis and subsequent venous thromboembolism in adults with trauma: A systematic review and meta-analysis
Source: Medicine (Baltimore). 2023 Oct 27;102(43):e35625. doi: 10.1097/MD.0000000000035625 (PMC10615543; doi:10.1097/MD.0000000000035625)

**Supplemental Digital Content Figure 2: Additional forest plots for sensitivity analysis, time to VTE diagnosis, LOS and ventilator days**


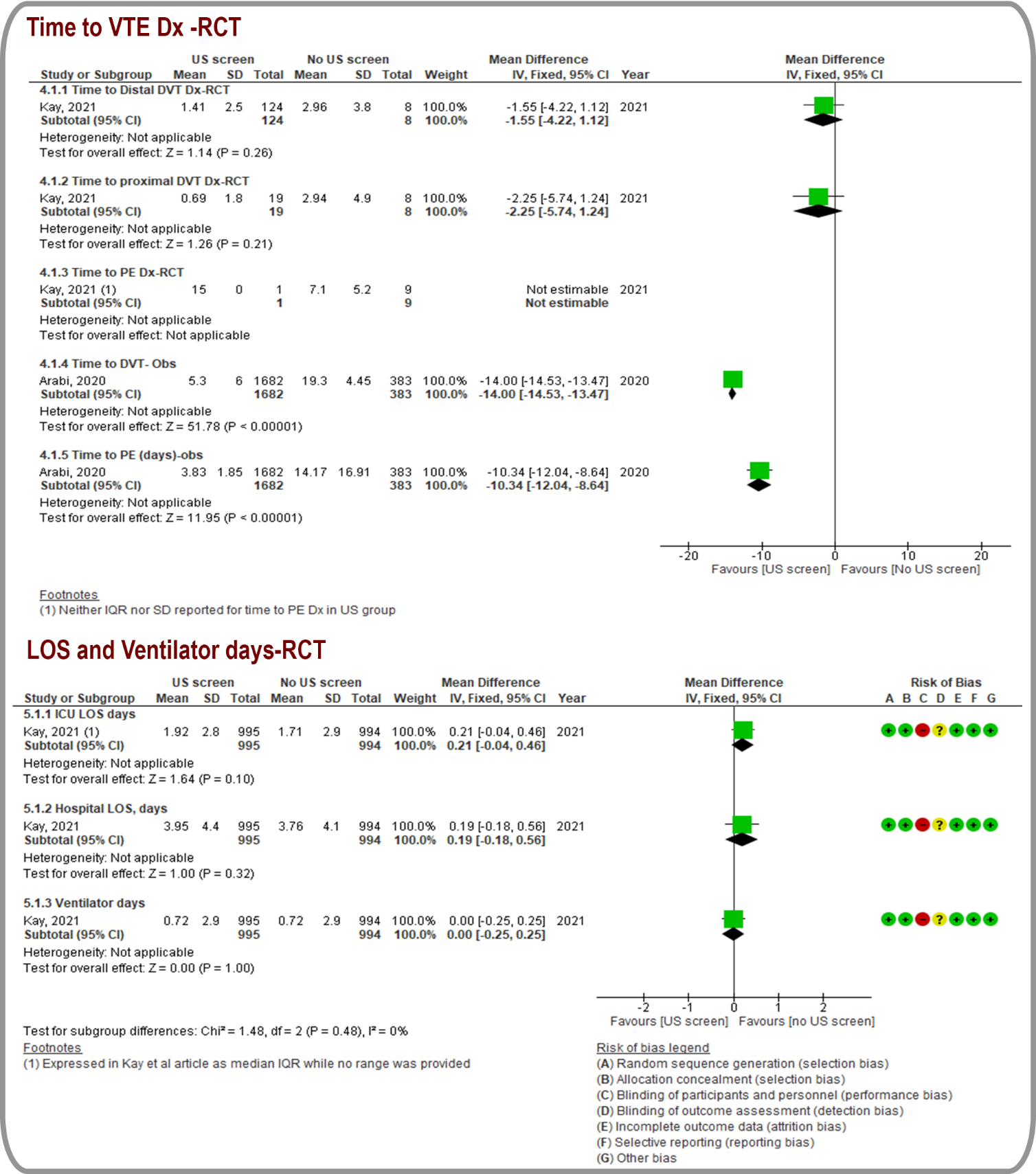

Supplement: Supplementary file 10 [file medi-102-e35625-s010.docx]
