# Supplementary material for: Ultrasound surveillance for deep venous thrombosis and subsequent venous thromboembolism in adults with trauma: A systematic review and meta-analysis
Source: Medicine (Baltimore). 2023 Oct 27;102(43):e35625. doi: 10.1097/MD.0000000000035625 (PMC10615543; doi:10.1097/MD.0000000000035625)
Supplement: Supplementary file 13 [file medi-102-e35625-s013.docx]

**Supplemental Digital Content Table 5: Recommendations from major societies on post-trauma thromboprophylaxis**

| **Society** | **Recommendation** |
| --- | --- |
| American Society of Haematology (ASH)^1^ | - For major trauma surgery, suggested pharmacological prophylaxis over no prophylaxis. - Did not make any specific recommendations regarding the timing, dose, or duration of pharmacological prophylaxis. |
| American College of Surgeons (ACS)^2^ | - Supported the use of STOP THE BLEED® kits to prevent bleeding in trauma patients. - Provided guidelines for the management of venous thromboembolism in surgical patients, which include risk assessment, mechanical and pharmacological prophylaxis, and treatment options. |
| Royal College of Surgeons (RCS)^3^ | - No specific guideline on post-trauma thromboprophylaxis, but it endorses the European guideline on management of major bleeding and coagulopathy following trauma , which recommends early and individualized administration of tranexamic acid, fibrinogen, prothrombin complex concentrate, and platelets. |
| World Federation of Societies of Anaesthesiologists (WFSA)^4,5^ | - No specific guideline on post-trauma thromboprophylaxis, but it supports the World Health Organization’s standards for safe surgery , which include measures to prevent venous thromboembolism in surgical patients. - Advocates for safe and affordable anaesthesia and pain relief for trauma patients. |
| The Western Trauma Association ^6^ | - All trauma patients should be assessed for their risk of VTE and bleeding on admission and daily thereafter. - Patients with low risk of VTE and bleeding should receive pharmacologic prophylaxis as soon as possible, preferably within 24 hours of admission. - Patients with high risk of VTE and low risk of bleeding should receive pharmacologic prophylaxis as soon as possible, preferably within 24 hours of admission, unless they have a contraindication such as active bleeding, intracranial haemorrhage, or spinal cord injury. - Patients with high risk of VTE and high risk of bleeding should receive mechanical prophylaxis until their bleeding risk decreases. They should also receive weekly screening for asymptomatic deep vein thrombosis (DVT) with routine ultrasound surveillance (RUSS) until they can receive pharmacologic prophylaxis. - Patients with contraindications to pharmacologic prophylaxis should receive mechanical prophylaxis and weekly RUSS indefinitely, unless their contraindications resolve, or they develop symptomatic DVT or pulmonary embolism (PE). |

**References:**

1. Anderson, D. R. et al. American Society of Hematology 2019 guidelines for management of venous thromboembolism: prevention of venous thromboembolism in surgical hospitalized patients. Blood Adv. 3, 3898–3944 (2019).

2. Yorkgitis BK, Berndtson AE, Cross A, et al. American association for the surgery of trauma/American college of surgeons-committee on trauma clinical protocol for inpatient venous thromboembolism prophylaxis after trauma. J Trauma Acute Care Surg. 2022;92(3):597–604.

3. Recommendations | Venous thromboembolism in over 16s: reducing the risk of hospital-acquired deep vein thrombosis or pulmonary embolism | Guidance | NICE.

4. Thromboprophylaxis Part I : WFSA - Resources. https://resources.wfsahq.org/atotw/thromboprophylaxis-part-i-anaesthesia-tutorial-of-the-week-223/.

5. Thromboprophylaxis Part 2 : WFSA - Resources. https://resources.wfsahq.org/atotw/thromboprophylaxis-part-2/.

6. Ley, E. J. et al. Updated guidelines to reduce venous thromboembolism in trauma patients: A Western Trauma Association critical decisions algorithm. J. Trauma Acute Care Surg. 89, 971–981 (2020).
